# Instrumental Assessment of a Chemical Skin Biostimulator Combining Ammonium Trichloroacetate and Homocysteic Acid in Photoaged Mature Skin: A Single-Arm Prospective Study

**DOI:** 10.3390/molecules31173038

**Published:** 2026-08-28

**Authors:** Anna Deda, Piotr Duda, Dominika Wcisło-Dziadecka, Adam Maszczyk, Sławomir Wilczyński

**Affiliations:** 1 Department of Practical Cosmetology and Skin Diagnostics, Faculty of Pharmaceutical Sciences in Sosnowiec, Medical University of Silesia in Katowice, ul. Jedności 8b, 41-200 Sosnowiec, Poland; 2 Institute of Biomedical Engineering, Faculty of Science and Technology, University of Silesia, 41-200 Sosnowiec, Poland; 3 Institute of Sport Sciences, The Jerzy Kukuczka Academy of Physical Education in Katowice, 40-065 Katowice, Poland; a.maszczyk@awf.katowice.pl; 4 Department of Basic Biomedical Sciences, Faculty of Pharmaceutical Sciences in Sosnowiec, Medical University of Silesia in Katowice, ul. Jedności 8b, 41-200 Sosnowiec, Poland; swilczynski@sum.edu.pl

**Keywords:** chemical skin biostimulator, photoaged facial skin, homocysteic acid (HoA), ammonium trichloroacetate (ATCA), grey-level co-occurrence matrix (GLCM), Cutometer, Antera 3D

## Abstract

Mature photoaged skin is characterised by concurrent deterioration of dermal extracellular matrix, impairment of the epidermal barrier and dysregulated melanogenesis. Chemical skin biostimulators that remodel the dermis without inducing overt exfoliation may offer a multimodal alternative to conventional peels. In this single-arm, prospective study, 25 women with Fitzpatrick phototype II and clinical signs of facial photoageing received four sessions of a homocysteic acid (HoA)-containing chemical biostimulator incorporating ammonium trichloroacetate (ATCA), and phytic and citric acids accompanied throughout by a standardised home-care regimen. Instrumental endpoints included viscoelasticity assessed by Cutometer, three-dimensional wrinkle volume and texture quantified by Antera 3D, grey-level co-occurrence matrix (GLCM) metrics for hyperpigmentation, and sebum and hydration measurements; tolerability was assessed by clinical inspection at each visit, although safety was not a pre-specified endpoint. All three Cutometer elasticity parameters increased by approximately 24% and remained significantly improved at 12 weeks, with a continued rise in biological elasticity (R7), a temporal pattern compatible with progressive dermal remodelling, although no histological verification was undertaken. Antera 3D documented a rapid (50–62%) reduction in wrinkle volume and texture roughness at 2 weeks, followed by a partial regression stabilising at 79–83% of maximal improvement at 12 weeks. In the hyperpigmented subgroup (*n* = 14), GLCM contrast decreased and homogeneity increased by 18–19%, indicating more uniform reflectance and reduced optical heterogeneity, with effects persisting at 12 weeks. Hydration improved and sebum secretion decreased; no serious adverse event was observed or reported, although adverse events were not recorded systematically. In this uncontrolled, single-arm study, a protocol based on a HoA-containing chemical skin biostimulator was associated with durable improvements in instrumentally assessed dermal elasticity and a partially reversible, yet sustained, enhancement of wrinkles, texture and hyperpigmentation in mature photoaged skin. The mechanisms proposed to underlie this profile—neocollagenesis and remodelling of the extracellular matrix, barrier restoration and modulation of melanogenesis—are interpretative rather than directly demonstrated. Because a combined commercial formulation was evaluated within a protocol that included a standardised home-care regimen, the observed changes cannot be attributed to any individual ingredient, including HoA, and the contribution of the home-care regimen cannot be separated from that of the in-office treatment. The absence of a control arm precludes causal attribution, and these findings—obtained in a small cohort of Fitzpatrick phototype II women, with a hyperpigmentation subgroup of only fourteen—should be regarded as hypothesis-generating.

## 1. Introduction

Mature skin is the result of the combined effects of chronological ageing and photoageing, with chronic exposure to UV radiation being responsible for the majority of clinically visible signs of skin ageing [1,2]. The skin ageing process involves both structural changes in the epidermis, dermis and vascular system, as well as mechanisms leading to hyperpigmentation, including melasma. With age, there is a gradual decline in keratinocyte proliferation, thickening of the stratum corneum and flattening of the dermo-epidermal junction, resulting in impaired barrier function, increased transepidermal water loss (TEWL) and greater susceptibility to environmental damage. At the same time, in the dermis, there is a reduction in the density and disorganisation of collagen and elastin fibres, a reduction in the levels of proteoglycans and hyaluronic acid, as well as a decline in fibroblast activity, which results in a loss of skin firmness, elasticity and regenerative capacity [1,2,3,4,5,6]. Clinically, photoageing manifests as dryness, roughness and a ‘pitted’ skin surface, deep wrinkles and furrows, laxity, uneven skin tone, telangiectasia and the presence of pigmentary changes, such as lentigines, which overlap with the endogenous ageing process in mature skin.

Acquired hyperpigmentation, including lentigines and post-inflammatory hyperpigmentation, constitutes one of the most visible signs of photoageing in mature skin, resulting from chronic, cumulative exposure to UV and visible light [1,7,8]. Melasma, in turn, is a chronic, often recurrent dyschromia with a multifactorial aetiology, involving the interaction of genetic and hormonal factors, exposure to UV/HEVL radiation, oxidative stress and local inflammation [6,9,10]. Recent data suggest that melasma is not merely a melanocyte dysfunction, but a disorder with characteristics of ‘photoageing’, in which solar elastosis, damage to the basement membrane, increased angiogenesis, endothelial cell proliferation and an increase in the number of mast cells in the upper dermis are observed. This view of melasma as a form of localised photoageing of the skin is significant for assessing the efficacy of biostimulants, which modify not only melanocyte activity but also the architecture of the extracellular matrix and the condition of collagen fibres [9,10,11]. The process of photoageing also involves a remodelling of the skin’s microcirculation, encompassing both angiogenesis and endothelial damage, which promotes the development of telangiectasia and persistent erythema, particularly on the face [3,12].

Current trends in anti-ageing and regenerative medicine are increasingly promoting treatments aimed at restructuring the skin without causing significant damage or severe inflammation. The aesthetic market is seeking treatments which, on the one hand, reduce the visible signs of skin ageing—wrinkles, loss of firmness, and hyperpigmentation—and, on the other hand, have a beneficial effect on improving the epidermal barrier, cellular repair mechanisms, whilst exhibiting strong antioxidant activity. Chemical skin stimulators are perfectly in line with current trends—they promote skin remodelling without the intense exfoliation characteristic of chemical peels.

Their mechanism of action differs from that of conventional chemical peels, as their primary aim is to modulate fibroblast activity, remodelling of the extracellular matrix and melanocyte activity, rather than to induce controlled damage and exfoliation of the epidermis [13,14]. In the case of many conventional trichloroacetic acid (TCA)-based peels, the therapeutic effect is associated with the induction of necrosis of the epidermis and/or the upper dermis, clinically visible as ‘frost’, followed by a process of re-epithelialisation and skin remodelling. Chemical biostimulators, on the other hand, are designed to stimulate fibroblasts to produce structural skin proteins, to remodel the extracellular matrix (ECM) of the dermis and, depending on the active ingredients, to influence the epidermal barrier, melanogenesis or the functions of the blood vessel endothelium, with minimal or no visible desquamation [15]. In addition to conventional TCA, commercial preparations also utilise its derivative—ammonium trichloroacetate (ATCA)—whose mechanism of action involves protein coagulation and the induction of controlled damage at the dermal level, which in turn stimulates fibroblasts to proliferate and increase the synthesis of collagen, elastin and other ECM components [14]. A recent development among chemical stimulators is the use of homocysteic acid (HoA), a novel patented antioxidant introduced into aesthetic dermatology as a biostimulating and photoprotective component, whose mechanisms of action are described in terms of the modulation of oxidative stress, methylation and transsulphuration pathways, and its effect on the metabolism of sulphur-containing amino acids [16,17,18]. Homocysteic acid (HoA) in the chemical skin biostimulator is proposed to act primarily as an endogenous NMDA receptor agonist, initiating the influx of calcium ions into epidermal cells and restoring physiological calcium-mitochondrial signalling pathways. In keratinocytes, the activation of NMDA receptors is associated with the regulation of the expression of differentiation proteins (including involucrin and filaggrin) and with enhanced proliferation and migration, which translates into faster re-epithelialisation, improved barrier integrity and anti-ageing potential through an indirect effect on the extracellular matrix. At the same time, modulation of these receptors influences calcium balance in the layers of the epidermis, reduces oxidative stress and supports the maintenance of epidermal homeostasis, which is important both in healing and in the prevention of photo-induced damage [16,17,19]. The mechanistic account outlined in this paragraph derives from in vitro and ex vivo studies of keratinocytes and melanocytes [16,17,18,19,20,21]; it is presented here as the rationale for the formulation under study and not as an established feature of the in vivo response to it.

In melanocytes, NMDA-dependent signalling regulates cell morphology, dendritic processes and the transfer of melanosomes to keratinocytes, whilst calcium influx enhances tyrosinase activity and influences the course of melanogenesis. Studies using NMDA agonists and antagonists indicate that activation of this pathway increases the number of filopodia and the efficiency of melanosome transfer, whilst receptor blockade impairs this process, alters the cytoskeleton and may reduce melanin synthesis whilst maintaining melanocyte survival [18]. In the context of chemical biostimulators, HoA is regarded as a regulator of melanocyte activity—at low concentrations, it supports physiological pigmentation and protection against oxidative stress, without excessive stimulation leading to hyperpigmentation [16,18]. This mechanism opens up the prospect of using NMDA modulation in the treatment of pigmentary disorders, with a potentially lower risk of irritation and post-inflammatory hyperpigmentation than with conventional depigmenting agents [16,17,18,19].

Although most data pertain to keratinocytes and melanocytes, it is suggested that NMDA signalling may also indirectly modulate fibroblast function by influencing the dynamics of the extracellular matrix, collagen remodelling and the interface between the epidermis and dermis. Maintaining normal calcium influx and mitochondrial activity in epidermal cells promotes the creation of a favourable microenvironment for fibroblasts, which may result in increased collagen synthesis, improved skin elasticity and better fibre organisation during healing [16,17,20].

The effect of HoA on the skin’s vascular microenvironment is also significant—by modulating NO/ROS pathways and angiogenic factors (particularly VEGF), it may limit abnormal angiogenesis and improve endothelial function [21], which is important for the telangiectasia and persistent erythema observed in photodamaged skin. In combination with ATCA, which acts on fibroblasts, and other antioxidant ingredients, a chemical skin-stimulating formulation may promote the regeneration of both the dermis and the epidermis [16,17,18,19,20,21]. The addition of phytic and citric acids in low concentrations exhibits a mild keratolytic effect—they facilitate the detachment of corneocytes by loosening corneodesmosomal junctions. Phytic acid is also a known chelator of transition metal ions (Fe^3+^, Cu^2+^), which limits Fenton reactions and reduces ROS production in UV-exposed skin, thereby exerting an antioxidant effect and supporting anti-hyperpigmentation therapies [22,23,24]. Citric acid, as a low-molecular-weight alpha-hydroxy acid (AHA), improves skin texture (smoothing, brightening) and may help to lighten superficial dyschromia, whilst initiating a mild regenerative signal in the living layers of the epidermis [25].

The state of the evidence concerning homocysteic acid in dermatology should be stated plainly, since it bears on how the present results may be interpreted. Homocysteic acid is an endogenous metabolite of the transsulphuration pathway and a well characterised endogenous agonist of the NMDA receptor, described in neuroscience since the 1980s [19]. Its use as a component of topical dermatological preparations is, by contrast, recent. At the time of writing, it is present, to our knowledge, in a single class of commercially available professional formulations positioned as chemical skin biostimulators, for which the proposed indications are facial photoageing, uneven skin tone and hyperpigmentation including melasma, enlarged pores and seborrhoea. The peer-reviewed clinical evidence consists of one small prospective pilot study in which the formulation was combined with microneedling [14]. The mechanistic account summarised above rests on in vitro and ex vivo studies of NMDA receptor signalling in keratinocytes [20] and melanocytes [18] in which homocysteic acid was not itself the agent investigated. The reported tolerability profile of this class of formulation comprises transient stinging on application and transient erythema, without the visible desquamation characteristic of conventional trichloroacetic acid peels, although the published clinical experience remains small. The present study should therefore be read as one of the first instrumental evaluations of a homocysteic acid-containing biostimulator, and its findings as hypothesis-generating rather than confirmatory.

The aim of this study was to evaluate, using non-invasive instrumental methods, the changes in photoaged facial skin observed after a four-session protocol employing a chemical biostimulator that combines ammonium trichloroacetate, homocysteic acid, phytic acid and citric acid, together with a standardised home-care regimen. Homocysteic acid is the component of principal interest, and is the one named in the title, because it is the only constituent of the formulation whose introduction into topical dermatology is recent and whose clinical evidence base is, as outlined above, confined to a single pilot study; ammonium trichloroacetate belongs to an established class of trichloroacetate-based biostimulators, and phytic and citric acids are long-established cosmetic actives present here at low concentration in a supporting role. Because the design was single-arm and uncontrolled, the study was intended to characterise and quantify the response of the treated cohort rather than to establish the efficacy of any individual component.

## 2. Results

### 2.1. Participants

**Figure 1:**
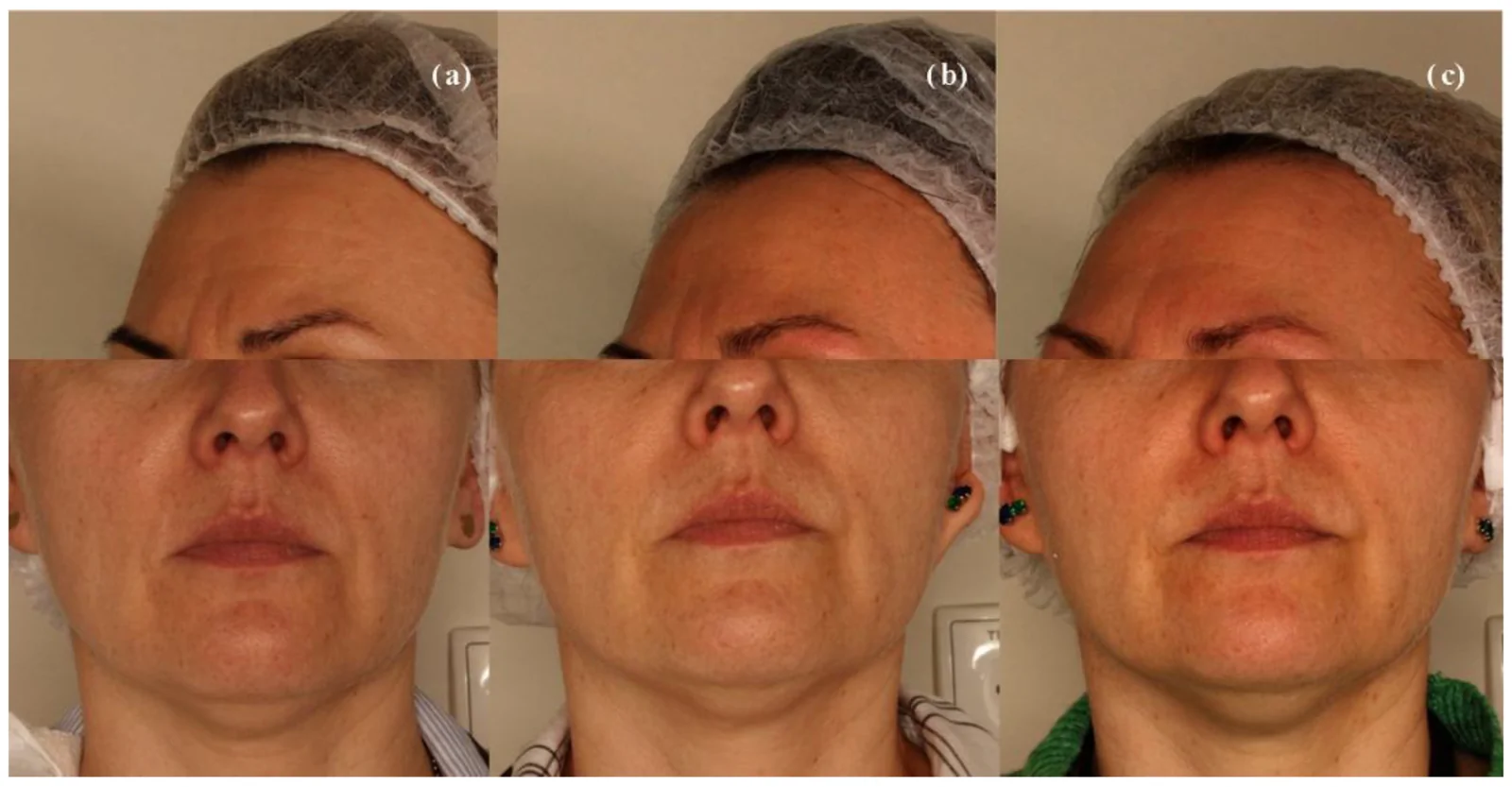
Oblique view of the forehead and frontal view of the lower face showing clinical outcome of chemical skin stimulation: (**a**) baseline before treatment; (**b**) 2 weeks after a series of four sessions; (**c**) 12 weeks after completion of the four-session treatment course.

Between 29 October 2025 and 1 April 2026, 27 volunteers were assessed for eligibility. Two were excluded before enrolment because of health-related contraindications, including active skin disease. The remaining 25 eligible women were enrolled, received all four scheduled treatment sessions, completed the baseline (T0), 2-week (T1) and 12-week (T2) assessments, and were included in the main analyses. No participant withdrew, discontinued treatment or was lost to follow-up. The participant flow is shown in Supplementary Figure S3. Twenty-five women completed all three assessments. All were of Fitzpatrick phototype II and all presented clinical signs of facial photoageing. Age ranged from 39 to 69 years (mean 52.8 ± 10.0 years; median 50 years, interquartile range 46 to 62); two participants were in their fourth decade, nine in their fifth, seven in their sixth and seven in their seventh. Fourteen participants (56%) presented with facial hyperpigmentation, of whom ten had melasma and four post-inflammatory hyperpigmentation; these fourteen constituted the subgroup in which the grey-level co-occurrence matrix analysis was performed. At baseline the cohort showed the viscoelastic and topographical profile expected of established photoageing: gross elasticity R2 was 0.703 ± 0.020 (median 0.709, range 0.671 to 0.733), net elasticity R5 0.723 ± 0.020 (median 0.729, range 0.690 to 0.753) and biological elasticity R7 0.403 ± 0.020 (median 0.413, range 0.375 to 0.427). Wrinkle volume was greatest in the nasolabial folds (10.86 ± 5.78 mm^3^; median 10.25, range 2.84 to 25.45) and lowest in the periorbital region (5.34 ± 2.34 mm^3^; median 4.38, range 2.34 to 11.83), with intermediate values for horizontal forehead lines (8.26 ± 3.19 mm^3^) and glabellar lines (6.25 ± 2.78 mm^3^). Surface micro-relief was correspondingly irregular, with a texture score of 58.9 ± 10.6, roughness Ra of 12.12 ± 2.24 micrometres and maximum height of 0.289 ± 0.071 mm. Stratum corneum hydration was 52.8 ± 8.5 arbitrary units and sebum output 120.7 ± 14.0 microgram/cm^2^. Baseline values for all endpoints are given in Table 1. All instrumental endpoints were available for the full cohort with the exception of the GLCM indices, which were quantified in the fourteen participants with hyperpigmentation. All participants showed clinical improvement in skin condition (Figure 1).

### 2.2. Skin Firmness (Cutometry)

**Figure 2:**
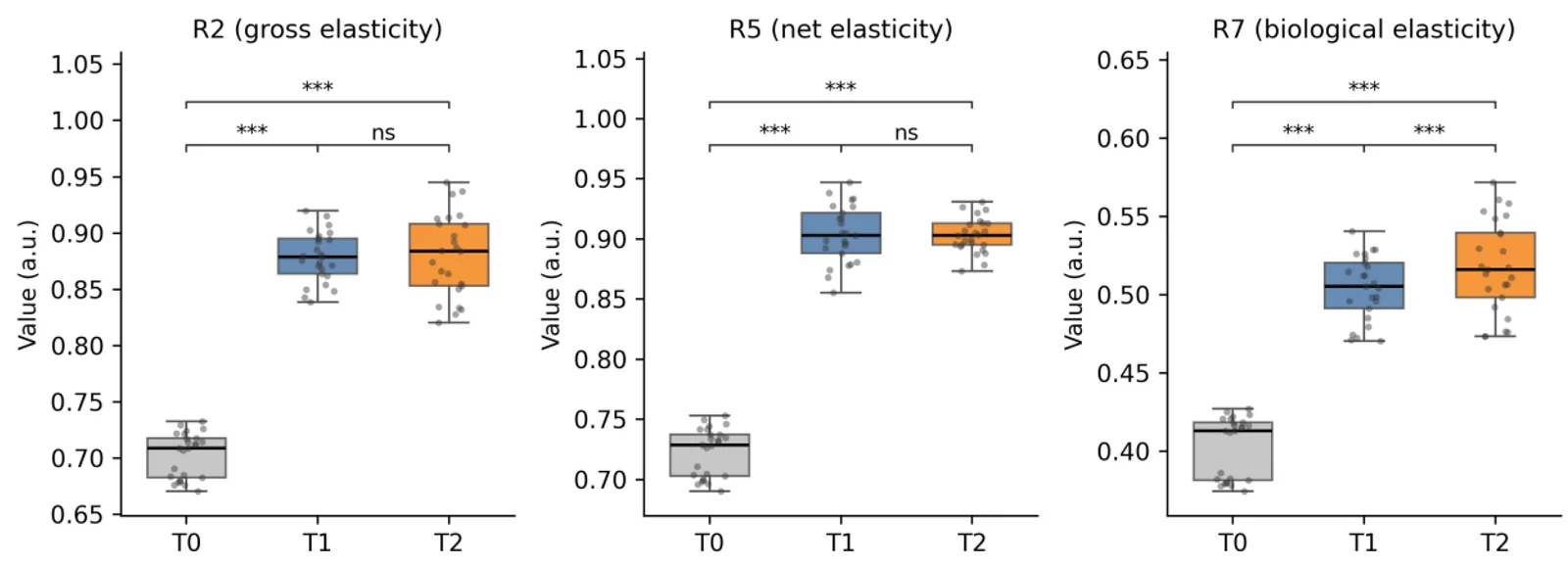
Cutometer viscoelastic parameters (R2, gross elasticity; R5, net elasticity; R7, biological elasticity) at baseline (T0), 2 weeks (T1), and 12 weeks (T2) after a series of four treatments. Grey boxes represent baseline values (T0), blue boxes represent values at 2 weeks (T1), and orange boxes represent values at 12 weeks (T2). Boxes denote the median and interquartile range, and whiskers the 1.5 × IQR range; individual participant values are overlaid (*n* = 25). Brackets indicate Holm-adjusted Wilcoxon signed-rank comparisons: ***p < 0.001, ns = not significant.

All three viscoelastic ratios increased markedly after the treatment series, and the improvement was preserved at 12 weeks (Table 2, Figure 2). Gross elasticity (R2) rose from a median of 0.709 at baseline to 0.879 at T1 (+23.9%) and 0.884 at T2 (+24.7%), with a significant effect of time (χ^2^(2) = 37.9, *p* < 0.001, W = 0.76). Net elasticity (R5) followed the same pattern (0.729 → 0.903 → 0.903; +23.9% at both post-treatment visits; χ^2^(2) = 37.9, *p* < 0.001, W = 0.76), as did biological elasticity (R7; 0.413 → 0.505 → 0.516; +24.9% at T2; χ^2^(2) = 49.5, *p* < 0.001, W = 0.99).

Pairwise comparisons showed large, significant gains from baseline to both T1 and T2, with every one of the 25 participants improving on each ratio (all Holm-adjusted *p* < 0.001, r = +1.00; Table 3). The T1–T2 contrast was non-significant for R2 and R5 (*p* = 0.909 and *p* = 0.841), indicating a stable effect between 2 and 12 weeks, whereas R7 improved further between the two post-treatment visits (*p* < 0.001, r = +1.00). Skin firmness therefore improved after treatment and was fully maintained, with R7 continuing to improve, up to 3 months. Because suction cutometry integrates the mechanical behaviour of the stratum corneum, the viable epidermis and the dermis, three exploratory post hoc analyses were performed to establish whether the viscoelastic gain could be accounted for by a change in stratum corneum hydration. The two variables were dissociated in time: at T1 the median R7 had risen by 22.3% while corneometric hydration was unchanged (+0.9%, *p* = 0.925). Within participants, the relative change in R7 was not correlated with the concurrent relative change in hydration over any interval (Spearman rho = +0.32, *p* = 0.116 for T0 to T1; rho = −0.03, *p* = 0.878 for T0 to T2; rho = −0.28, *p* = 0.171 for T1 to T2; *n* = 25). Finally, the elasticity parameters diverged between T1 and T2: R7 increased further in 24 of 25 participants, whereas R2 and R5 plateaued (12 of 25 each), indicating a selective gain in the elastically recovered fraction of the deformation rather than a uniform change in distensibility of the kind expected from an increase in bulk water content. These analyses were not pre-specified and are reported without correction for multiplicity.

Values are medians with interquartile range in brackets. R_a_, arithmetic roughness; χ^2^(2), Friedman statistic with 2 degrees of freedom; *W*, Kendall’s coefficient of concordance (effect size). GLCM indices were obtained in 14 participants—all other parameters in 25.

Δ%, percentage change in the median from baseline. Pairwise cells report the Holm-adjusted *p*-value with the matched-pairs rank-biserial correlation (*r*) in parentheses; positive *r* indicates a higher parameter value at the later time point. For parameters where higher values are desirable (elasticity ratios, hydration, tone homogeneity), improvement corresponds to positive *r*; for the remaining parameters improvement corresponds to negative *r*.

### 2.3. Wrinkle Volume (Antera 3D)

**Figure 3:**
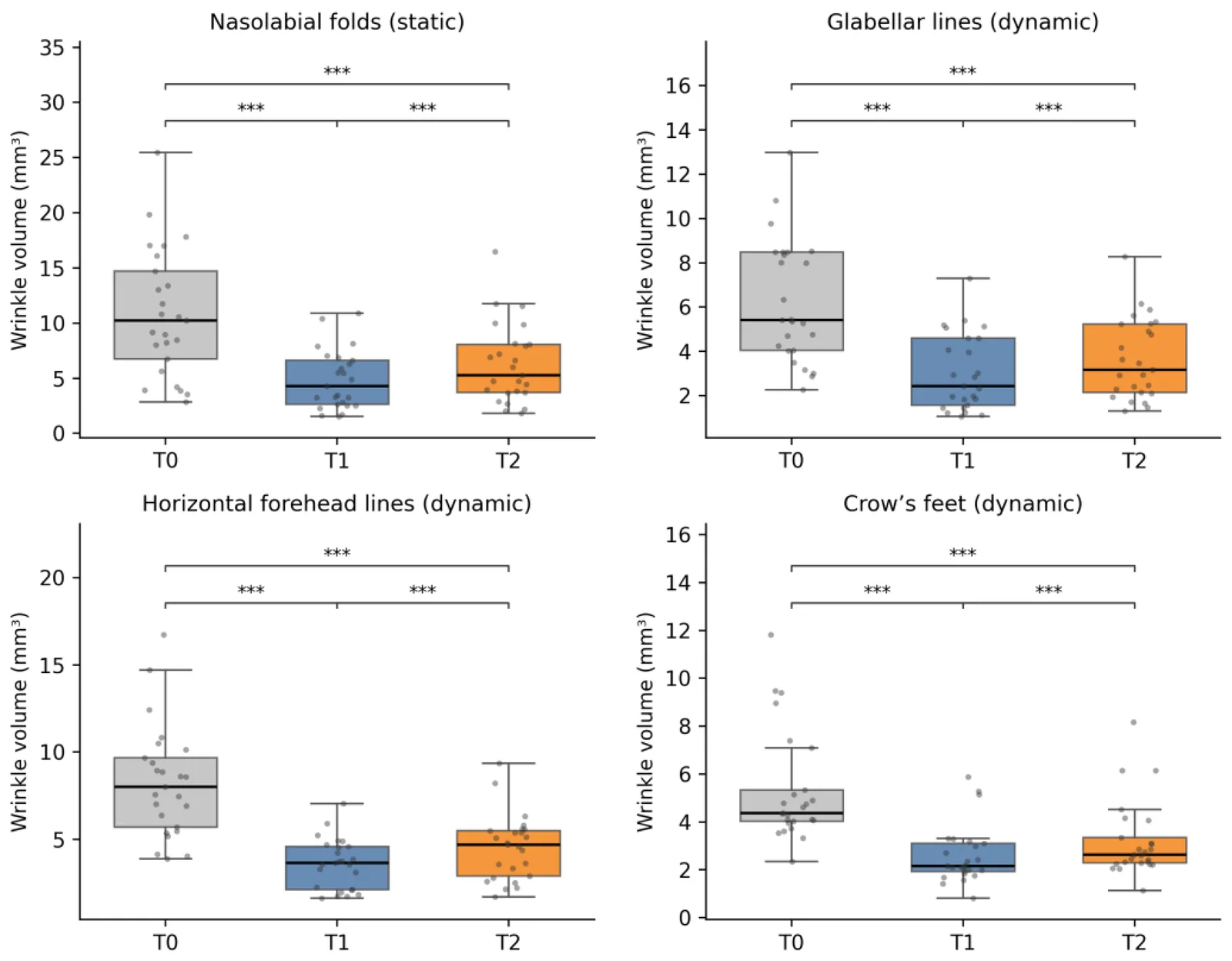
Wrinkle volume (Antera 3D) by facial region at T0, T1, and T2 (*n* = 25). Grey boxes represent baseline values (T0), blue boxes represent values at 2 weeks (T1), and orange boxes represent values at 12 weeks (T2). Nasolabial folds are a predominantly static (gravitational) region; glabellar lines, horizontal forehead lines, and crow’s feet are dynamic (mimetic) regions. Boxes show median and IQR with overlaid individual values; brackets denote Holm-adjusted Wilcoxon signed-rank comparisons (*** *p* < 0.001).

**Figure 4:**
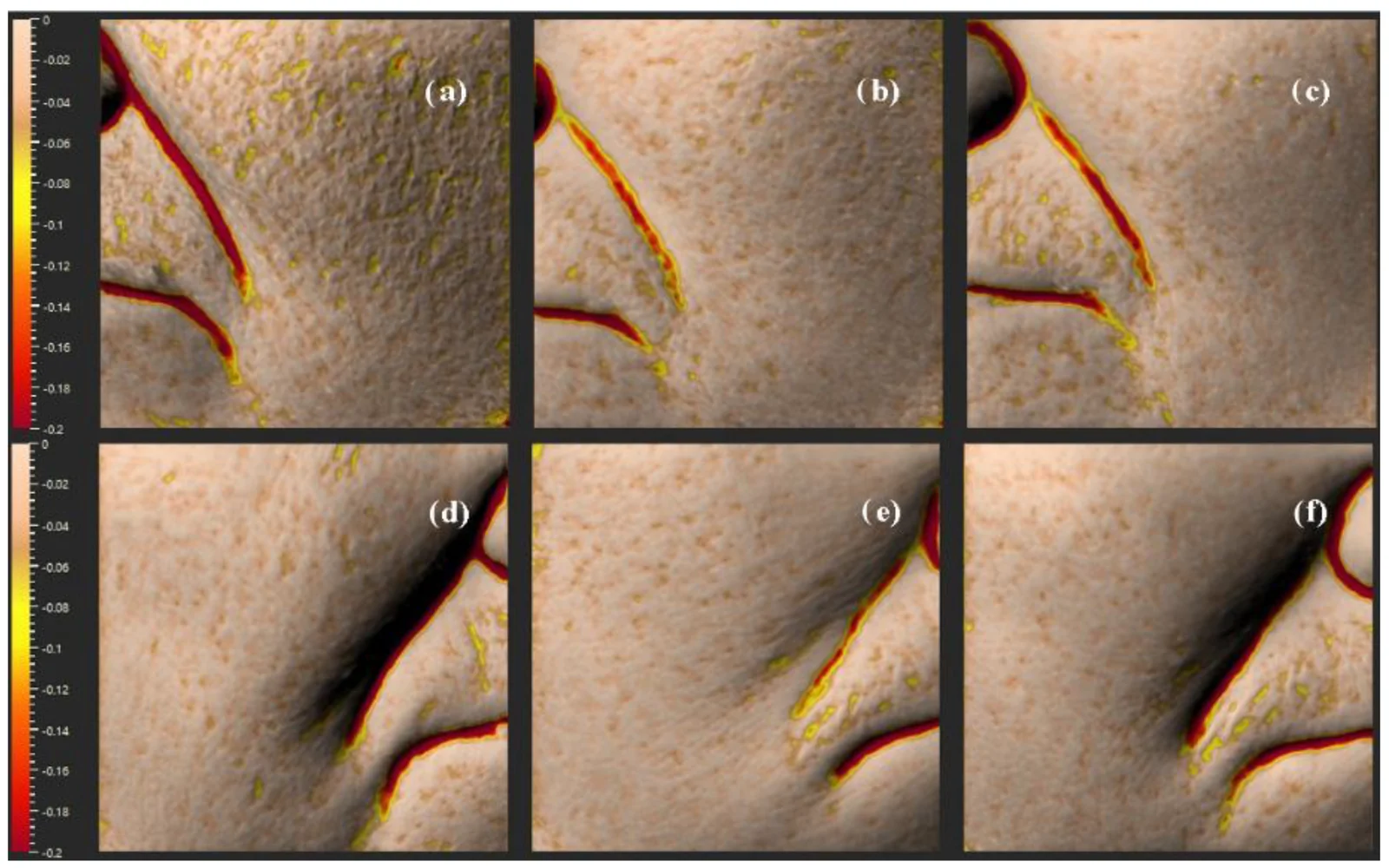
Antera 3D wrinkle volume maps of the nasolabial region showing the effect of chemical skin stimulation: (**a**,**d**) baseline before treatment; (**b**,**e**) 2 weeks after a series of four sessions; (**c**,**f**) 12 weeks after completion of the four-session treatment course.

All four wrinkle regions showed a large reduction in volume 2 weeks after the treatment series, followed by a partial rebound at 12 weeks that nevertheless left every region significantly below baseline (Table 2 and Table 3, Figure 3). Nasolabial fold volume decreased from 10.25 mm^3^ to 4.27 mm^3^ at T1 (−58.3%) and 5.26 mm^3^ at T2 (−48.7%); glabellar lines from 5.41 to 2.43 (−55.1%) and 3.17 mm^3^ (−41.4%); horizontal forehead lines from 8.02 to 3.65 (−54.5%) and 4.68 mm^3^ (−41.7%); and crow’s feet from 4.38 to 2.16 (−50.6%) and 2.63 mm^3^ (−39.8%). An example of the reduction in the depth of nasolabial folds, as measured using the Antera 3D device, is shown in Figure 4.

The effect of time was significant for every region (Friedman *p* < 0.001; *W* ≥ 0.96). Baseline-to-T1 and baseline-to-T2 reductions were all significant with maximal effect sizes (Holm-adjusted *p* < 0.001; *r* = −1.00), while the T1–T2 contrast was significant and positive for all regions (*p* < 0.001; *r* ≥ +0.99), confirming a measurable partial regression of the surface relief between 2 and 12 weeks.

### 2.4. Skin Texture (Antera 3D)

**Figure 5:**
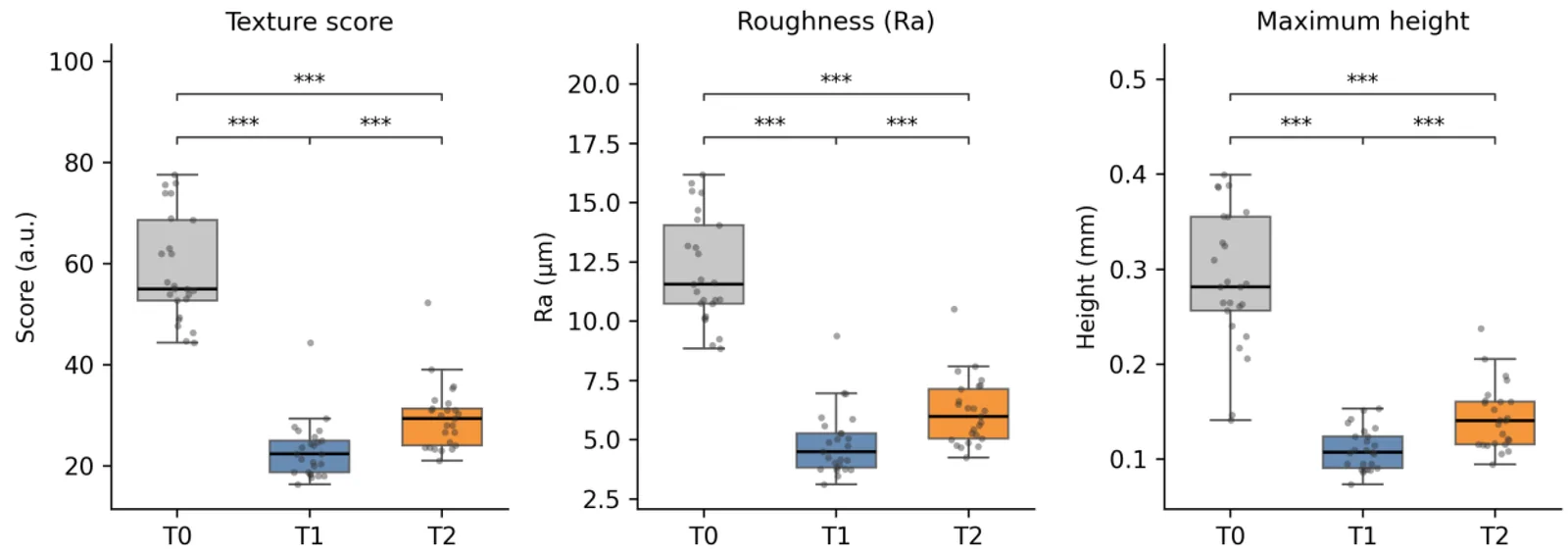
Antera 3D skin-texture indices—texture score, roughness R_a_, and maximum height—at T0, T1, and T2 (*n* = 25). Grey boxes represent baseline values (T0), blue boxes represent values at 2 weeks (T1), and orange boxes represent values at 12 weeks (T2). Lower values indicate a smoother surface. Boxes show median and IQR with overlaid individual values; brackets denote Holm-adjusted Wilcoxon signed-rank comparisons (*** *p* < 0.001).

**Figure 6:**
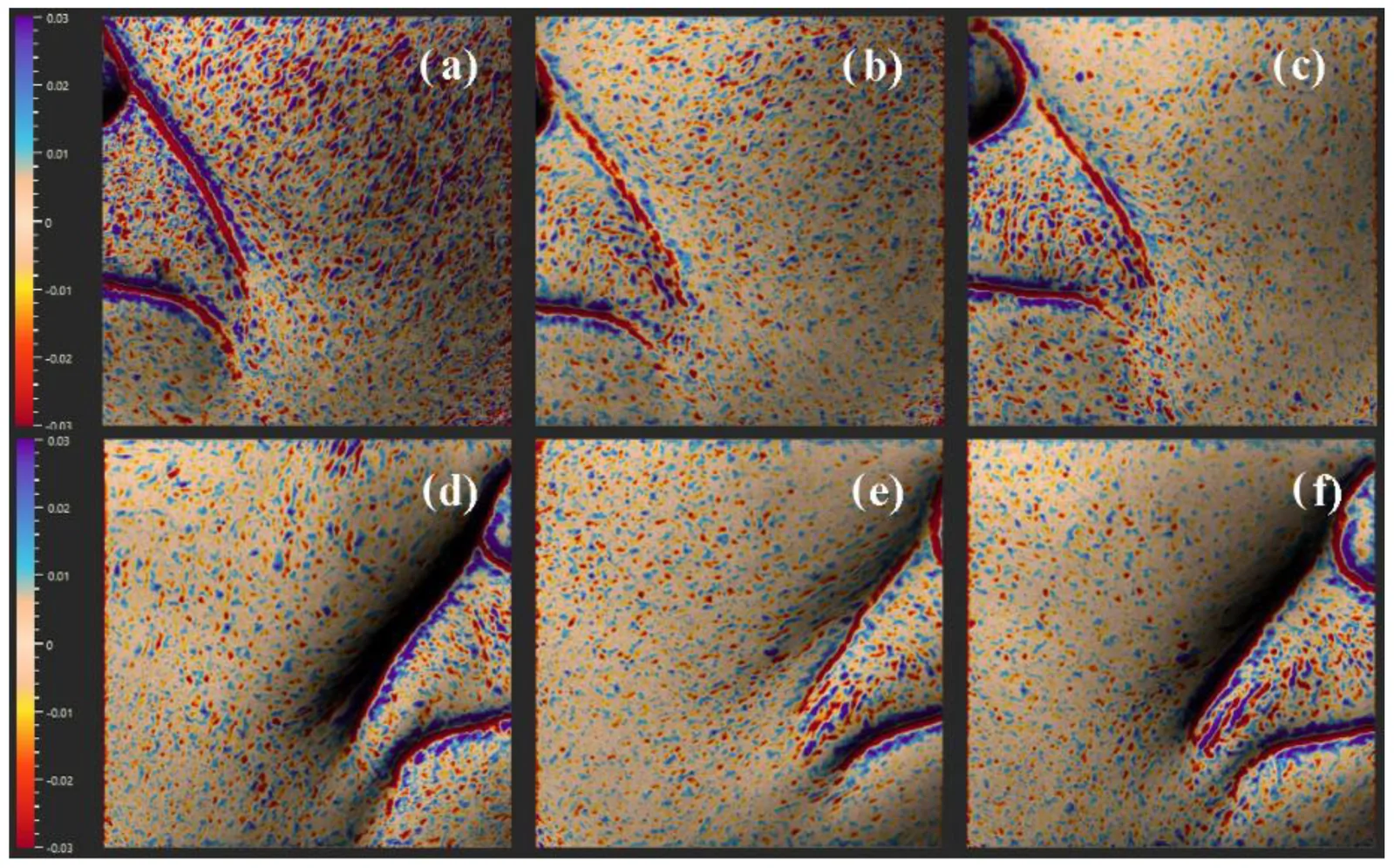
Antera 3D texture maps of the nasolabial region showing the effect of chemical skin stimulation: (**a**,**d**) baseline texture before treatment; (**b**,**e**) texture 2 weeks after a series of four sessions; (**c**,**f**) texture 12 weeks after completion of the four-session treatment course.

The three texture indices improved substantially at T1 and, like wrinkle volume, regressed partially by T2 while remaining well below baseline (Table 2, Figure 5). An example of an improvement in skin texture, as measured by the Antera 3D device, is shown in Figure 6.

The texture score fell from 55.0 to 22.3 (−59.4%) at T1 and 29.3 (−46.7%) at T2; roughness R_a_ from 11.57 to 4.51 µm (−61.0%) and 5.99 µm (−48.2%); and maximum height from 0.282 to 0.107 mm (−61.9%) and 0.141 mm (−50.1%). All omnibus tests were significant (χ^2^(2) = 50.0, *p* < 0.001, *W* = 1.00), and every baseline and T1–T2 contrast reached significance (Holm-adjusted *p* < 0.001; |*r*| = 1.00; Table 3).

### 2.5. Durability of the Surface Response

For the parameters that peaked at 2 weeks, participants retained a median of 79–83% of the peak improvement at 12 weeks (the detailed durability data for wrinkle volume and texture parameters are provided in Supplementary Table S4), corresponding to a partial regression of approximately 17–21%. Retention was comparable across wrinkle regions (nasolabial 79%, glabellar 81%, horizontal forehead 83%, crow’s feet 79%) and texture indices (82–83%). Contrary to the a priori expectation, the retained effect for the static nasolabial folds (median 79%) did not differ significantly from that of the pooled dynamic regions (median 80%; Wilcoxon signed-rank *p* = 0.19, *r* = −0.30). The partial regression of the surface topography over 3 months was therefore of similar relative magnitude for dynamic (mimetic) and static wrinkles.

### 2.6. Skin Hydration and Sebum

**Figure 7:**
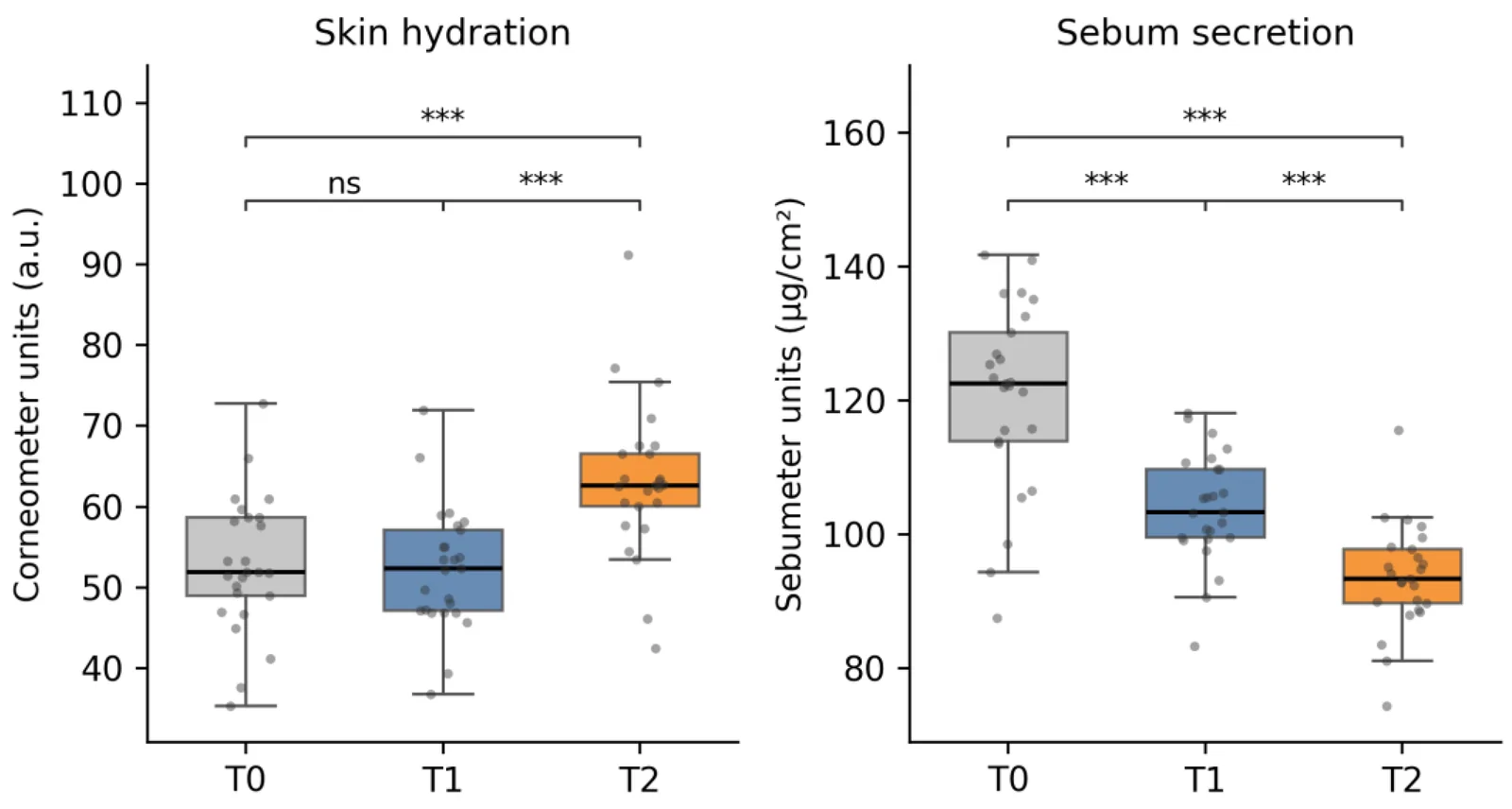
Skin hydration (corneometry, arbitrary units) and sebum secretion (sebumetry, µg/cm^2^) at T0, T1, and T2 (*n* = 25). Grey boxes represent baseline values (T0), blue boxes represent values at 2 weeks (T1), and orange boxes represent values at 12 weeks (T2). Boxes show median and IQR with overlaid individual values; brackets denote Holm-adjusted Wilcoxon signed-rank comparisons (*** *p* < 0.001, ns = not significant).

Corneometric hydration was essentially unchanged 2 weeks after treatment (51.9 → 52.4; +0.9%; T0–T1 *p* = 0.925) but increased significantly by 12 weeks (62.6; +20.7% versus baseline; T0–T2 and T1–T2 *p* < 0.001, *r* = +1.00), indicating a delayed but robust hydration benefit (Table 2 and Table 3, Figure 7). Sebum secretion declined progressively across the three visits, from 122.6 to 103.4 (−15.7%) at T1 and 93.4 (−23.8%) at T2, with all pairwise contrasts significant (Holm-adjusted *p* ≤ 0.001; *r* ≤ −0.93), consistent with a sustained, cumulative reduction in sebaceous activity.

### 2.7. Skin Tone Uniformity (GLCM)

**Figure 8:**
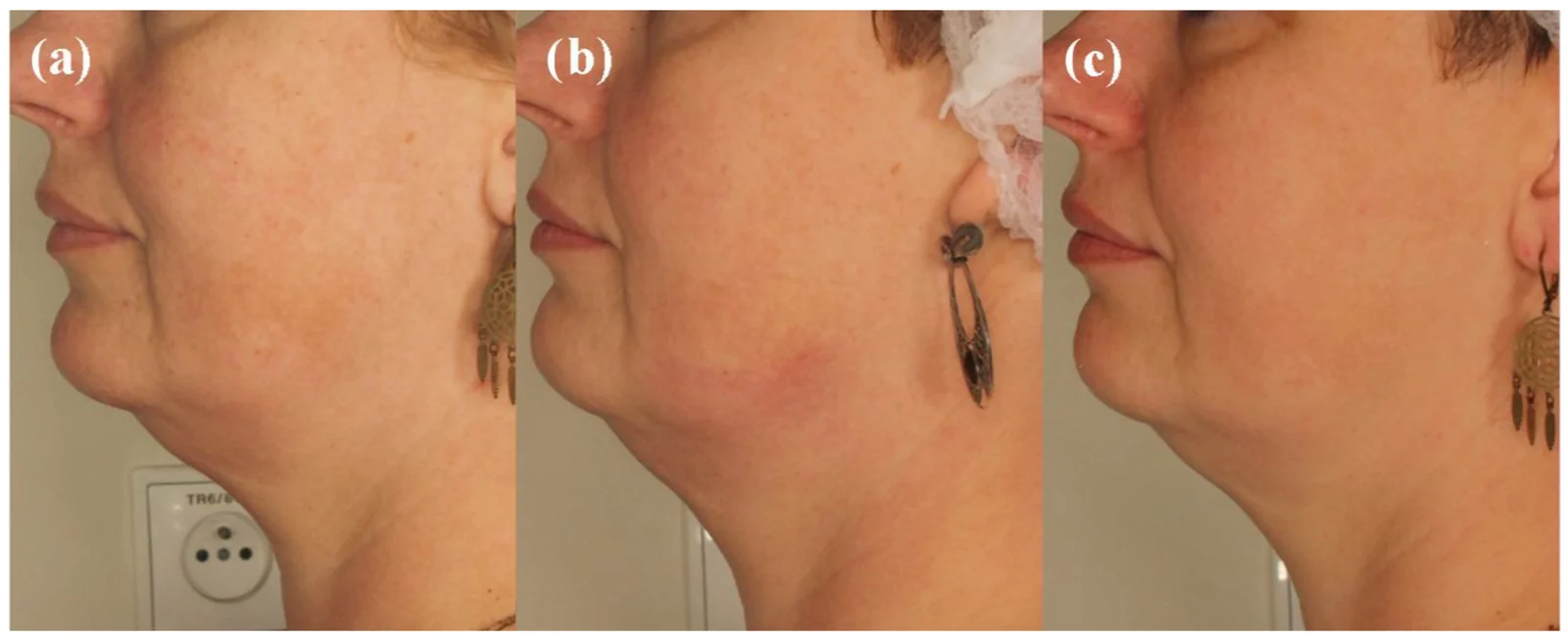
Lateral view of the lower face demonstrating reduction in facial hyperpigmentation following chemical skin stimulation: (**a**) baseline pigmentation pattern before treatment; (**b**) 2 weeks after a series of four sessions; (**c**) 12 weeks after completion of the four-session treatment course, showing more uniform skin tone and decreased mottled hyperpigmentation.

**Figure 9:**
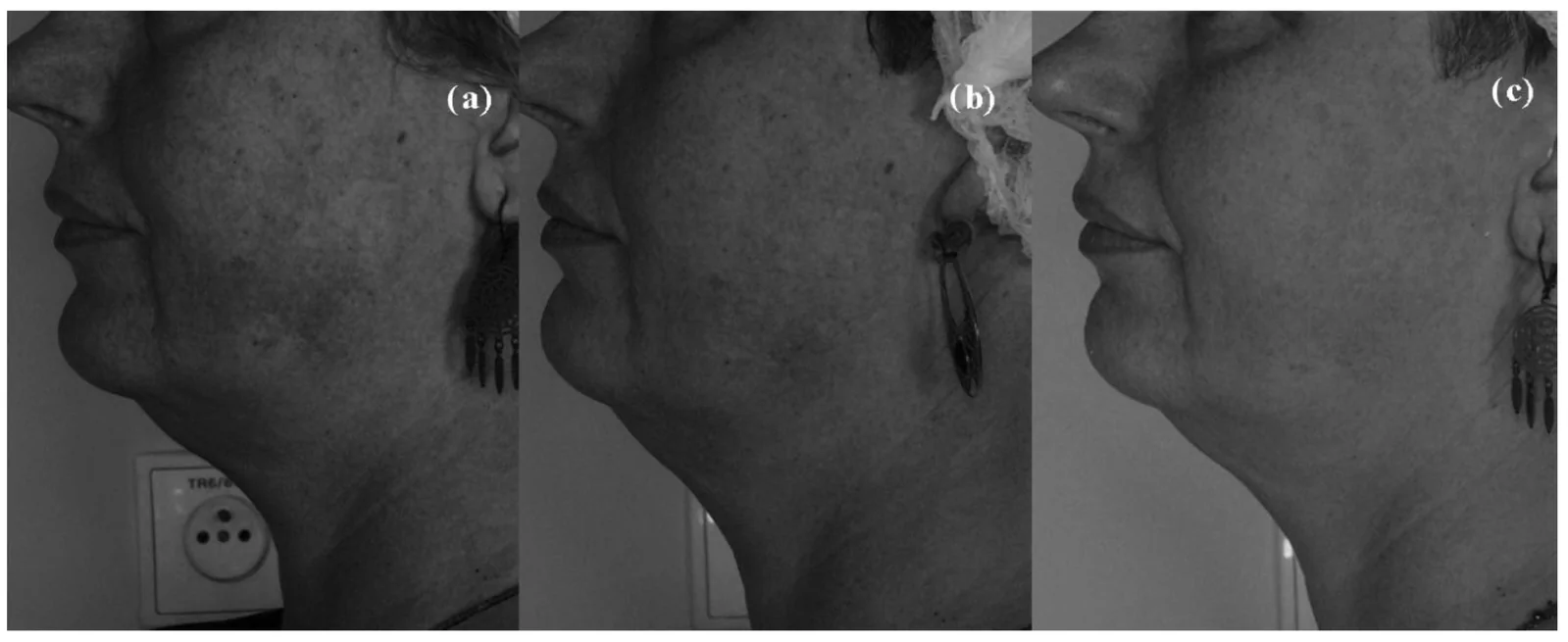
Ultraviolet photography of the lateral face illustrating changes in subclinical hyperpigmentation following chemical skin stimulation: (**a**) baseline distribution of UV-visible hyperpigmented macules before treatment; (**b**) 2 weeks after a series of four sessions; (**c**) 12 weeks after completion of the four-session treatment course, showing marked reduction and homogenisation of UV-detectable pigmentation.

All 14 female volunteers affected by hyperpigmentation showed a visible reduction in discolouration (Figure 8). Hyperpigmented lesions are particularly clearly visible under UV light; therefore, Figure 9 shows the reduction in hyperpigmentation following the procedure.

**Figure 10:**
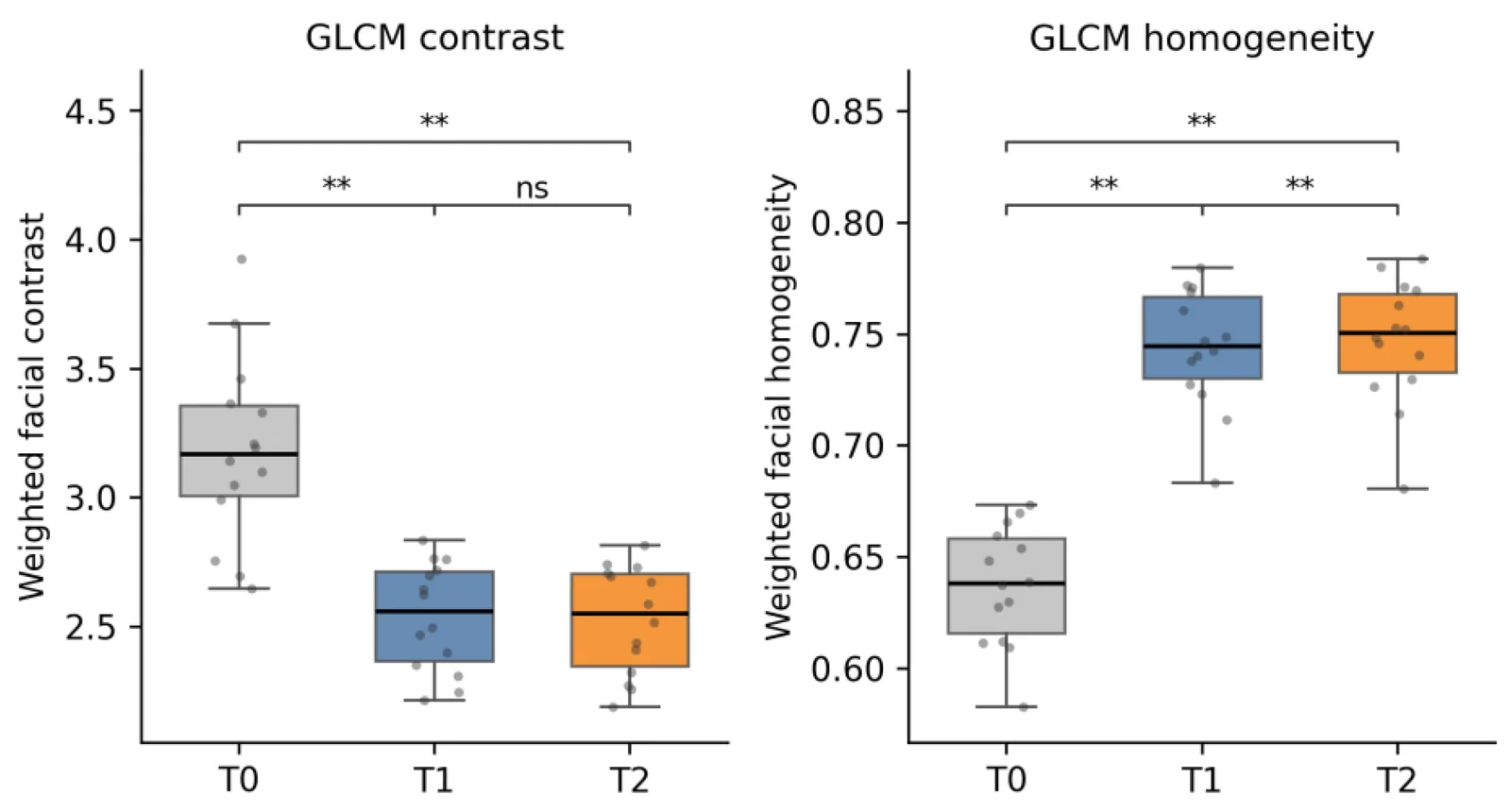
Weighted whole-face GLCM contrast and homogeneity at T0, T1, and T2 (*n* = 14). Grey boxes represent baseline values (T0), blue boxes represent values at 2 weeks (T1), and orange boxes represent values at 12 weeks (T2). Lower contrast and higher homogeneity indicate a more uniform skin tone. Boxes show median and IQR with overlaid individual values; brackets denote Holm-adjusted Wilcoxon signed-rank comparisons (** *p* < 0.01, ns = not significant).

In the 14 participants with GLCM data (volunteers with hyperpigmentation), the weighted facial contrast decreased (3.17 → 2.56 → 2.55; −19.5% at T2) and homogeneity increased (0.638 → 0.745 → 0.750; +17.6% at T2), both indicating a more even surface reflectance consistent with reduced hyperpigmentation (Table 2, Figure 10). The effect of time was significant for contrast (χ^2^(2) = 21.6, *p* < 0.001, *W* = 0.77) and homogeneity (χ^2^(2) = 24.6, *p* < 0.001, *W* = 0.88). Improvements from baseline were significant at both post-treatment visits (Holm-adjusted *p* ≤ 0.003; |*r*| = 1.00). Between 2 and 12 weeks, contrast remained stable (*p* = 0.064) and homogeneity improved slightly further (*p* = 0.003), indicating a fully maintained tone benefit.

Taken together, the instrumental measurements demonstrated a broad improvement in skin condition after the treatment series that was largely maintained at 12 weeks. Skin firmness, hydration, sebum regulation, and tone uniformity showed no regression—indeed, hydration and sebum continued to improve—whereas the surface-topography parameters (wrinkle volume and texture) peaked at 2 weeks and then regressed partially, retaining roughly four-fifths of their peak improvement at 3 months.

### 2.8. Subjective (Self-Reported) Assessment of Treatment Effects

The post-treatment questionnaire was completed at the T1 visit, two weeks after the final session, and therefore corresponds in time to the instrumental measurements obtained at that visit; it was not repeated at T2.

To capture the subjective dimension of treatment outcomes, participants completed a structured questionnaire (Supplementary Files S2 and S3) designed to assess their personal perception of the effects of the chemical skin stimulation.

Subjective evaluation paralleled the instrumental findings: all 25 participants reported an overall improvement in skin condition and were satisfied with the treatment, and the proportion of women satisfied with the appearance of their face and neck rose from 48% (12/25) at baseline to 76% (19/25) after the series. On the −5 to +5 change scales, participants perceived significant improvements in facial appearance (median +4.2), wrinkle depth (+3.6) and firmness (+3.4; all *p* < 0.001 versus “no change”; Table 4 and Figure S1 included in the Supplementary Material), with high satisfaction ratings for the wrinkle-reduction (median 8.2/10) and firming (7.9/10) effects.

### 2.9. Influence of Age

Because the cohort spanned three decades, the relationship between age and both baseline severity and treatment response was examined in an exploratory post hoc analysis. Age was strongly associated with baseline values: all three viscoelastic ratios declined with age (Spearman rho = −0.776, −0.770 and −0.776 for R2, R5 and R7; all Holm-adjusted *p* < 0.0001), whereas wrinkle volume and surface roughness increased (rho = +0.814 for glabellar lines, +0.711 for horizontal forehead lines, +0.553 for crow’s feet, +0.737 for texture score, +0.784 for roughness Ra and +0.683 for maximum height; all Holm-adjusted *p* < 0.02; nasolabial folds rho = +0.458, Holm-adjusted *p* = 0.064). Hydration and sebum output were unrelated to age (rho = −0.109 and +0.048; Holm-adjusted *p* = 1.00). The relative change from baseline at 12 weeks, by contrast, was associated with age for only one of the twelve endpoints, net elasticity R5 (rho = +0.690; Holm-adjusted *p* = 0.0016); wrinkle volume in all four regions and all three texture indices were unrelated to age. For R7 the association with relative change (rho = +0.525 at T1) was not reproduced for the absolute change (rho = +0.235, *p* = 0.259), indicating a floor effect arising from lower baseline values in older participants rather than a greater biological response; for R5 at T2 both the absolute and the relative change correlated with age (rho = +0.658 and +0.690). When the cohort was divided at the median age of 50 years, median relative changes at 12 weeks were comparable between strata for wrinkle volume (nasolabial folds −41.4% below 50 years versus −41.0% at or above 50 years, *p* = 0.722; glabellar lines −42.9% versus −42.6%, *p* = 0.978) and for texture (texture score −50.0% versus −51.6%, *p* = 0.180), and every participant in both strata improved on every viscoelastic, topographical and textural parameter (11 of 11 and 14 of 14). Age therefore determined baseline severity strongly but influenced the magnitude of the response minimally, and in the direction of greater relative benefit in older participants.

### 2.10. Tolerability

Across 100 treatment sessions involving 25 participants, the only treatment-related events were a transient burning sensation during application and transient redness that subsided within 2–3 h after treatment. No visible peeling, crusting, or post-inflammatory hyperpigmentation was observed, no serious adverse events occurred, and no participants discontinued treatment or withdrew from the study. Because safety was not a pre-specified endpoint and adverse events were not recorded on a structured form (Section 4.12), these observations characterise the tolerability of the protocol as applied and do not constitute a formal safety assessment.

## 3. Discussion

In this single-arm, prospective study, four sessions using a chemical skin biostimulator combining ammonium trichloroacetate (ATCA), homocysteic acid (HoA), phytic acid and citric acid, were followed by broad, statistically significant and largely sustained improvements in five independent, measurable parameters of photoaged mature skin: viscoelasticity, wrinkle volume, surface micro-relief, hydration of the stratum corneum and sebum secretion, as well as uniformity of skin tone. Friedman’s omnibus tests demonstrated statistical significance for each endpoint, with large effect sizes (Kendall’s W ≥ 0.75), and for most surface parameters—maximum effect sizes for matched pairs in paired comparisons (|r| = 1.00). The convergence of five methodologically distinct measurement principles—optical 3D reconstruction, suction cutometry, capacitive corneometry, photometric sebometry and image analysis based on a grey-level co-occurrence matrix (GLCM)—in a consistent pattern of improvement argues against measurement artefacts and indicates a genuine, multidirectional change in skin condition, although the uncontrolled design does not permit that change to be attributed causally to the intervention. Such a multimodal pattern is what one would expect from a formulation designed to act on the main molecular pathways of skin ageing—remodelling of the extracellular matrix (ECM), restoration of the barrier and hydration, control of oxidative stress, and regulation of melanogenesis—for which its ingredients were selected [1,2,3,4,5,6]; this correspondence is, however, interpretative: the single-arm design does not demonstrate any of these mechanisms, and the observed changes cannot be apportioned between the formulation, its individual components and the concomitant home-care regimen.

### 3.1. Restoration of Viscoelastic Properties Is Consistent with the Remodelling of the Skin Matrix

The increase in skin firmness was the finding that provided the most insight into the possible mechanisms involved. All three Cutometer indices increased by approximately 24 per cent, and, most importantly, this improvement was fully maintained after 12 weeks, with biological elasticity (R7) continuing to increase significantly between the 2-week and 12-week visits. R7 reflects the ratio of elastic to total deformation and is considered the parameter most closely linked to the integrity and organisation of the dermal fibre network, rather than to transient surface hydration. Its continuous increase over three months is difficult to explain by epidermal or oedematous effects; rather, it would be consistent with progressive neocollagenesis and reorganisation of the elastic fibre scaffold, although suction cutometry cannot identify the tissue substrate of the change and this interpretation therefore remains hypothetical—a process whose time course (ranging from weeks to months) corresponds to the kinetics of fibroblast-driven matrix synthesis [4,5]. This interpretation is consistent with the presumed mechanism of action of ATCA, a trichloroacetic acid salt formulated to reach the papillary dermis and the upper reticular dermis without causing superficial coagulation, thereby stimulating fibroblasts to synthesise type I and III collagen and to remodel fragmented elastic fibres [13,14,15]. A similar, sustained increase in elasticity parameters measured by a Cutometer was observed in the study by Augustyniak and Rotsztejn [26], in which a five-session course of non-ablative radiofrequency treatment of the periorbital area in 23 patients led to a significant increase in R2 and R6 (*p* < 0.0001) and a clinical reduction in wrinkles of approximately 33 per cent, which was interpreted as an improvement in skin tension in response to the gradual remodelling of the extracellular matrix. In a 12-week prospective study by Sadick and Harth [27], using a home-use, multi-source RF device for facial rejuvenation, a significant improvement in facial skin firmness and elasticity was demonstrated, confirmed by both clinical assessment and measurements using the MPA 580 Cutometer, alongside an increase in skin collagen content. In turn, in a more recent study of HIFU targeting periorbital, perioral and neck wrinkles, the mean R7 value measured by the Cutometer was significantly higher after 10 weeks than at baseline, and the improvement persisted for at least up to the 16th week of follow-up, alongside favourable changes in the assessment of surface topography and the global scale of aesthetic improvement [28]. A comparison of these data with our results highlights that the time courses of the viscoelastic response of the dermis observed here are similar to those of interventions with a deep focal point and the ability to induce collagen remodelling, such as radiofrequency or HIFU, distinguishing the present response pattern from typical superficial procedures with a predominantly epidermal effect. At the same time, the sustained 12-week increase in R7 observed in our patients following a series of four treatments using a chemical skin biostimulator, with R2 and R5 reaching a plateau, indicates a selective enhancement of the elastic component relative to total deformability, a pattern that would be expected from progressive neocollagenesis and reorganisation of elastic fibres, and that is less readily explained by a transient increase in tension from fibre contraction under the influence of heat or tissue oedema, as observed following some RF and HIFU treatments [26,27,28].

### 3.2. A Two-Component Model of Wrinkle and Texture Response

Wrinkle volume and the three Antera 3D texture indices exhibited a different pattern of change to firmness [29]. Each of these measures fell sharply after 2 weeks (by approximately 50–62 per cent), and then partially reversed by week 12, whilst remaining 40–50 per cent below baseline and showing highly statistically significant differences in every comparison. Participants retained a median of 79–83% of their maximum improvement, corresponding to a repeatable partial reversal of changes of approximately 17–21%, which was remarkably consistent across all areas and measures. This pattern is best explained by a two-component response: an early, partially reversible component—likely reflecting transient post-treatment oedema, corneocyte condensation, and acute changes in hydration and light scattering induced by alpha-hydroxy acids (citric, phytic and, in home care, glycolic acid) acting on the stratum corneum [22,23,24,25]—superimposed upon a slower, persistent dermal component, which would be consistent with tissue matrix remodelling, although the present design permits neither the demonstration of that process nor its attribution to any individual component of the formulation. A similar biphasic pattern of superficial and deep responses has been described in studies using Antera 3D to assess the outcomes of rejuvenation treatments [30]. Dou et al. [31], in a prospective, randomised split-face trial comparing micro-needle radiofrequency (MFR) with a non-ablative 1565 nm fractional laser (NAFL) in patients with facial photoageing, observed a significant improvement in facial skin laxity, wrinkles and pores, as well as a reduction in nasolabial fold volume, as assessed by Antera 3D and VISIA analysis after three treatment sessions; the most significant changes in surface topography occurred in the first few weeks following treatment and subsequently stabilised during further follow-up.

Contrary to our a priori hypothesis, the durability of the surface response did not differ between predominantly static (gravitational) nasolabial folds and dynamic (expressive) areas; the relative regression was of a similar magnitude in both cases. If the lasting effect depended mainly on the mechanical dynamics of repeated muscle contractions, one would expect greater regression of dynamic wrinkles. Their similar behaviour is compatible with a lasting effect that is structural rather than mechanically modulated, that is, one arising from a change common to all facial zones rather than from regional muscle activity. We emphasise that dermal matrix remodelling is offered here as a hypothesis consistent with the observed pattern and not as a demonstrated mechanism: the present study provides no histological, immunohistochemical or imaging evidence of matrix change, and alternative explanations, including a persistent alteration of stratum corneum properties or of the optical characteristics of the skin surface, cannot be excluded from the data reported. This is a clinically useful observation, as it suggests that the intervals between maintenance treatments can be planned uniformly for all facial zones, rather than being tailored to the type of wrinkle.

The contribution of the home-care regimen to this biphasic pattern warrants separate consideration, since the nightly fluid contained glycolic acid at low concentration and alpha-hydroxy acids are known to improve surface texture. The regimen was, however, applied identically and without interruption by all participants from the first in-office session until the final assessment, and was therefore constant across the observation period, whereas the in-office sessions ended before the two-week assessment. A surface improvement driven principally by continued nightly exfoliation would be expected to persist or to accumulate with ongoing use. The observed pattern is the reverse: between two and twelve weeks, with home care uninterrupted, all three texture indices deteriorated in 25 of 25 participants and wrinkle volume increased in all four regions in 23 of 25, while biological elasticity continued to increase in 24 of 25. The kinetics of the surface response are thus aligned with the in-office sessions rather than with the continuing home regimen. This observation does not exclude a contribution of the home-care products to the absolute magnitude of the improvement, which cannot be isolated in the present design, but it argues against their being the principal determinant of either the early peak or the subsequent partial regression.

### 3.3. The Delayed Increase in Hydration Is More Consistent with Restoration of the Barrier and Matrix than with Surface Hydration

The course of hydration was instructive precisely because of this delay. Corneometric capacity remained unchanged after 2 weeks and then increased by approximately 21% after 12 weeks. One would expect a purely topical moisturising effect of glycerine or acids to manifest early and then subside; therefore, the opposite temporal pattern—a delayed onset without regression—suggests a regenerative rather than a cosmetic mechanism. Two complementary processes are possible. Firstly, the replenishment of the dermal matrix (proteoglycans and hyaluronic acid) and improved fibre organisation enhance the skin’s water-binding capacity over the same time horizon of several weeks as the increase in viscoelastic properties [4,5]. Secondly, at the level of the epidermis, it is suggested that HoA acts as an endogenous NMDA receptor agonist, which restores physiological calcium signalling in keratinocytes and increases the expression of differentiation proteins, such as filaggrin and involucrin, thereby improving the efficiency of the epidermal barrier and reducing transepidermal water loss [16,17,19,20]. A parallel, progressive decrease in sebum secretion (−16% after 2 weeks, worsening to −24% after 12 weeks) indicates a cumulative normalisation of sebaceous gland activity and is consistent with the gradual restoration of epidermal homeostasis, rather than acute damage leading to lipid loss. Both accounts are interpretative: neither transepidermal water loss nor any epidermal or dermal biomarker was measured, and a cumulative contribution of the standardised home-care regimen—in particular the daily emollient cream—to the delayed hydration gain cannot be excluded in a design without a home-care-only comparison.

### 3.4. Uniformity of Skin Tone and a Hypothetical Role of NMDA-Dependent Regulation of Melanogenesis

In 14 participants with discolouration, the GLCM contrast decreased and homogeneity increased by approximately 18–19 per cent, indicating both a more uniform surface reflectance and a reduction in the optical heterogeneity characteristic of dyschromia; this benefit was fully maintained, and homogeneity improved slightly further between weeks 2 and 12. From a mechanistic point of view, this is consistent with the current understanding of melasma and sunspots as localised manifestations of photoageing in which pigmentary changes are associated with solar elastosis, damage to the basement membrane and dermal remodelling, rather than solely with melanocyte hyperactivity [6,9,10,11]. It should be emphasised that no NMDA-related biomarker was measured in the present study, so that the receptor-mediated account offered here is an interpretive framework drawn from the cited in vitro literature rather than an inference supported by data reported in this paper. Within this framework, a formulation that simultaneously remodelled the extracellular matrix (ECM) and modulated melanogenesis could plausibly improve skin tone. At the molecular level, in vitro studies indicate that NMDA receptor signalling can regulate the branching of melanocyte dendrites and the transfer of melanosomes in a physiological and self-limiting manner, suggesting the possibility of controlled pigmentation with a potentially lower risk of irritation and post-inflammatory hyperpigmentation than with conventional depigmenting agents [16,18]; whether HoA engages this pathway in the treated skin was not examined in the present study. Phytic acid provides an orthogonal antioxidant mechanism: as a chelator of Fe^3+^ and Cu^2+^, it limits the generation of reactive oxygen species via the Fenton reaction in skin exposed to UV radiation, thereby suppressing the oxidative and inflammatory response leading to melanogenesis [22,23,24]. The persistence of the improvement in tone uniformity at twelve weeks is of interest given the well-known tendency of melasma to recur. Its interpretation is, however, constrained in a way that the present design cannot overcome. All fourteen participants with hyperpigmentation applied, twice daily and throughout the observation period, a brightening serum containing niacinamide and tranexamic acid together with ferulic acid, alpha-lipoic acid, lactoferrin and phytic acid. Niacinamide and tranexamic acid are among the best-documented topical depigmenting agents in melasma, and a regimen of this composition would be expected to improve optical uniformity in its own right, in the absence of any in-office procedure. With fourteen participants, no control arm and no serum-free comparison, the contribution of the biostimulator and that of the serum to the observed reduction in GLCM contrast and increase in homogeneity cannot be separated, and no estimate of their relative magnitude is possible from these data. The findings are therefore reported as the response of hyperpigmented skin to the combined regimen. The NMDA-dependent regulation of melanocyte dendricity and melanosome transfer described in vitro [16,18] remains one plausible contributor to that response, as does the antioxidant and metal-chelating action of phytic acid [22,23,24], but the present study provides no basis for attributing the effect preferentially to any of them. A comparison of the biostimulator with and without the concomitant serum, in a controlled design, would be required to do so. Our observations are consistent with the results of the study by Wawrzyk-Bochenek et al. [32], in which the GLCM method was used to quantitatively assess the efficacy of microneedle mesotherapy with 12% ascorbic acid in reducing hyperpigmentation on the forearm skin. The authors demonstrated an average increase in skin brightness of 10.6%, a decrease in GLCM contrast of 10.7% and an increase in homogeneity of 14.5%, with the greatest differences observed in the B channel of the RGB colour space, reflecting the particular sensitivity of this band to changes in melanin. As in our study, the simultaneous decrease in contrast and increase in uniformity were interpreted as a more even distribution of pigment and a reduction in the visibility of dyschromia, whilst the use of GLCM was considered a significant complement to traditional, subjective clinical scales. A comparison of the two studies highlights that texture analysis based on a grey-level co-occurrence matrix is sensitive to changes induced both by protocols hypothesised to act at the dermal level (here, the biostimulator-based protocol) and by predominantly epidermal procedures involving the delivery of vitamin C via microneedling. At the same time, the greater percentage decrease in contrast and increase in homogeneity observed in our study suggest that an integrated effect on the ECM and melanogenesis may lead to a more pronounced smoothing of the brightness map than monotherapies aimed solely at lightening the epidermis. This supports the usefulness of GLCM as a tool for differentiating the response profiles of various depigmentation protocols. More broadly, these changes may be viewed in the context of the skin as a neuro-immuno-endocrine organ, in which epithelial, immune, neural, vascular and endocrine-like signalling pathways jointly regulate barrier homeostasis, pigmentation, inflammation and extracellular-matrix remodelling. The present study did not evaluate these mediators directly; therefore, this framework is offered only as a biologically plausible context for the multimodal instrumental response observed after treatment [33].

### 3.5. Strengths and Limitations

The main advantages of this study are: multimodal, operator-independent equipment under standard imaging conditions, a predefined analytical plan, and statistical rigour: a uniform non-parametric framework, an ‘omnibus-then-contrasts’ structure with family-wise error rate control using the Holm–Bonferroni method, the reporting of effect sizes alongside *p*-values, and a persistence analysis for each participant, which quantifies rather than merely confirms the persistence of the effect. Nevertheless, the interpretation of the results should be treated with some caution due to several limitations. Firstly, and most importantly, the study was single-arm, uncontrolled, unrandomized and unblinded. No vehicle-treated, untreated or split-face control was included and no sham procedure was performed. The design therefore does not permit causal attribution of the observed changes to the intervention: the passage of time and the natural cycle of cutaneous repair, seasonal variation in photodamage, regression to the mean, the standardised home-care regimen applied throughout the observation period, and participant expectation all remain formally uncontrolled alternative explanations that the present data cannot exclude. Three features of the dataset make a purely non-specific explanation less likely: the magnitude of the changes, approximately 24% for all three viscoelastic ratios and 40 to 58% for wrinkle volume, in a cohort aged 39 to 69 years in whom the expected twelve-week natural trajectory of these parameters is stable or slowly deteriorating; the homogeneity of the response, with improvement in every participant on every viscoelastic and surface parameter; and the divergent, parameter-specific time courses described above, which a uniform time-related or expectation-related drift would not be expected to reproduce. These observations, however, constitute arguments of plausibility rather than evidence of causation, and the results reported here should be read as hypothesis-generating. Secondly, the biostimulator used in the clinic was part of a comprehensive treatment regimen, which included a regenerating cream, a glycolic acid lotion for night-time use, daily SPF 50+ sun protection and, in cases of hyperpigmentation, an active brightening serum; the effects described here are therefore those of the entire protocol, and the specific contribution of individual active ingredients—in particular the new HoA ingredient—cannot be isolated. The home-care regimen itself may have contributed materially to the observed outcomes: the daily emollient cream and the nightly 8% glycolic acid fluid could plausibly account for part of the delayed hydration gain and of the surface-texture improvement, and, in the hyperpigmentation subgroup, the niacinamide- and tranexamic acid-containing serum for part of the tone improvement. The findings should therefore be read as the response to the protocol as a whole rather than to the in-office formulation alone. Thirdly, the cohort was small (*n* = 25; *n* = 14 for GLCM), consisted exclusively of women and was restricted to Fitzpatrick phototype II, which limits the generalisability of the results to men, darker phototypes and other conditions associated with hyperpigmentation. The hyperpigmentation findings in particular rest on a subgroup of only fourteen participants (ten with melasma, four with post-inflammatory hyperpigmentation) and should be regarded as preliminary; their applicability to larger and more diverse populations, including the darker phototypes in which dyschromia is most prevalent, remains to be established. Fourthly, the follow-up period lasted three months, so the long-term durability of skin remodelling—and the ultimate course of partially reversible surface parameters—remains to be determined. Fifthly, the results were instrumental in nature and based on imaging; no histological or immunohistochemical confirmation of neocollagenesis or elastic fibre reorganisation was obtained, so the proposed cutaneous mechanisms, whilst consistent with the temporal course of the data, remain speculative. Sixthly, although the full quantitative composition of the formulation is disclosed in the patent covering the product and is reproduced in Table 5 so that the intervention can be independently replicated, the study evaluated the complete formulation within a standardised treatment protocol: no study arm omitted any component; the observed changes therefore cannot be apportioned among the individual actives—in particular, none of them can be attributed to homocysteic acid—and the dose–response relationship of any component remains undefined. The composition reported is, moreover, the nominal manufacturing composition disclosed in the patent, and the content of the actives in the batches used was not independently verified by the investigators. The mechanistic attributions offered in the Section 3 are accordingly qualitative and interpretative. Seventhly, self-reported outcomes were captured on a single occasion, at two weeks, that is, at the point of maximal instrumental improvement in surface topography, and the questionnaire was not repeated at twelve weeks; whether the partial regression of wrinkle volume and texture observed between two and twelve weeks is perceptible to participants therefore remains unknown, and the subjective data should not be read as evidence of a maintained subjective benefit at three months. Eighthly, safety was not a pre-specified endpoint and adverse events were not recorded on a structured form, so the absence of serious adverse events reported here rests on clinical inspection and spontaneous reporting rather than on systematic ascertainment. Finally, the cohort spanned three decades of age, and although the exploratory analysis reported in Section 2.9 found the magnitude of the response to be largely independent of age, a sample of 25 provides limited power for subgroup analysis; the stratified comparisons were performed in groups of 11 and 14 participants and were not corrected for multiplicity, so the apparent independence of the response from age should be regarded as reassuring rather than as established.

### 3.6. Directions for Further Research

These results justify the conduct of a randomised, controlled and—ideally—a ‘split-face’ study, in which a biostimulator containing HoA is compared with an established reference preparation for bioremodelling and with a vehicle, under standardised home care conditions, so that the additional contribution of the active substance administered in the clinic can be quantified. The inclusion of histological and immunohistochemical endpoints (collagen I/III, elastin, procollagen markers), a longer follow-up period to map the full temporal course of regression in surface parameters, and the recruitment of patients from a wider range of phototypes and of both sexes would significantly strengthen the evidence base. Mechanistic verification of the HoA-NMDA axis should be pursued directly rather than inferred, and can be specified concretely. Expression of the obligatory NMDA receptor subunit GluN1 (GRIN1) and of GluN2 subunits in keratinocytes and melanocytes, together with intracellular calcium flux measured with Fura-2 or Fluo-4 under blockade with MK-801 or D-AP5, would establish whether the pathway is engaged and whether the response is receptor-dependent. The epidermal differentiation proteins filaggrin and involucrin represent the proposed downstream read-out of the barrier effect and are accessible with minimal invasiveness through sequential tape stripping or confocal Raman spectroscopic profiling of natural moisturising factor. Matrix turnover could be indexed non-destructively by procollagen type I and type III propeptides (PICP, PIIINP) together with MMP-1 and TIMP-1 in suction blister fluid and, where biopsy is ethically justified, by immunohistochemistry for collagen I and III and for elastin. Tyrosinase, TYRP1 and melanosome transfer would address the melanogenic arm, and VEGF together with markers of oxidative stress would address the vascular and antioxidant components. Such a programme, ideally comparing the HoA-containing formulation with an otherwise identical HoA-free vehicle, would be required before the NMDA-dependent mechanism proposed here could be regarded as established in vivo. Formal tolerability assessments, by investigator-graded erythema and desquamation scales, together with participant-completed visual analogue scales, should be incorporated into the same study.

## 4. Materials and Methods

### 4.1. Study Participants

The study enrolled 25 female volunteers with Fitzpatrick phototype II, aged 39 to 69 years (mean 52.76 ± 9.95), all presenting clinical signs of photoaged skin. Fourteen volunteers had hyperpigmentation, of whom 10 were diagnosed with melasma and 4 with post-inflammatory hyperpigmentation. Exclusion criteria included pregnancy or breastfeeding; ongoing hormone therapy; systemic steroid use within three months prior to inclusion; topical retinoid use within one month and oral retinoid use within six months prior to treatment; chemical peel procedures within one month; application of arbutin and/or hydroquinone within three months; laser-based treatments within three months; as well as active inflammatory dermatoses, autoimmune diseases, and renal impairment. The protocol was approved by the institutional Bioethics Committee of the Medical University of Silesia (KNW/0022/KB1/27/16). The study is reported in accordance with the TREND statement for non-randomised evaluations of interventions [35]; the completed 22-item checklist is provided as Supplementary File S1. Items relating to randomisation, allocation concealment, blinding and comparison conditions are reported as not applicable, since the study was single-arm and uncontrolled.

### 4.2. Treatment Areas and Timing of Assessments

The biostimulation protocol was applied to predefined facial areas showing clinically visible signs of photoageing, including fine to moderate wrinkles, surface roughness, and irregular micro-relief. For quantitative assessment, measurements were focused on standardised regions of interest (ROIs), including the forehead (frown lines), forehead lines, cheeks (nasolabial folds) and periorbital areas (crow’s feet), which are particularly susceptible to UV damage and are commonly treated in aesthetic practices.

Skin assessments were carried out at three time points: T0, at baseline, prior to the first application of the biostimulator; T1, two weeks after the fourth and final application; and T2, twelve weeks after the fourth and final application.

At each visit, three-dimensional images were acquired before product application to avoid interference from transient erythema or surface shine. The same regions of interest (ROIs) were assessed at T0, T1 and T2, with particular attention paid to reproducing head position, facial expression and camera alignment, and to capturing the same wrinkle segments along their full length.

### 4.3. Procedure for Chemical Skin Stimulation

The chemical skin stimulation protocol was carried out four times in total to address clinical signs of photoageing, in accordance with the manufacturer’s guidelines and using a formulation containing ammonium trichloroacetate, homocysteic acid, phytic acid and citric acid (PRX plus, WiQO, Trieste, Italy). The composition of the in-office preparation, in descending order of concentration as declared by the manufacturer, is: Aqua, Ammonium Trichloroacetate, Glycerin, Homocysteic Acid, Acrylamide/Sodium Acryloyldimethyltaurate Copolymer, C13-14 Isoalkane, Citric Acid, Laureth-7, Phytic Acid, Tartaric Acid; the formulation pH is approximately 2. The full qualitative and quantitative composition of the formulation, which is disclosed in Example 7 of international patent application WO 2020/260199 A1 covering the product [34], is provided in Section 3.5 (Table 5). The exact weight-per-weight concentration of each component is proprietary to the manufacturer and was not disclosed to the investigators, who had no role in the development or manufacture of the product. This constraint is addressed in Section 3.5. Participants also received a standardised home-care regimen comprising a regenerating cream with a high shea butter content for daytime use and a smoothing night fluid containing glycolic acid at a concentration of 8% together with phytic and citric acids. All participants applied the same home-care regimen, without interruption and without modification, from the day of the first in-office session until the final assessment at twelve weeks; adherence was verified by a structured interview at each visit. The complete INCI listings of the home-care preparations are given in Supplementary Table S3. In addition, participants presenting with hyperpigmentation were provided with a lightening serum containing niacinamide, tranexamic acid, lactoferrin, ferulic acid, alpha-lipoic acid and phytic acid (Lightening serum, WiQo, Trieste, Italy), with instructions to apply it twice daily exclusively to areas affected by hyperpigmented lesions. Its complete INCI listing is given in Supplementary Table S3.

Following initial cleansing and degreasing with a solution formulated to lower the skin’s pH, three consecutive layers of the product containing ammonium trichloroacetate, homocysteic acid, phytic acid and citric acid were applied as the first step in the procedure. Each layer was massaged into predefined facial areas (forehead, nose, left and right cheeks, chin, and upper lip area), and the subsequent layer was applied only after the previous one had been fully absorbed—defined operationally as massaging until the surface of the glove became dry. Once three layers had been applied, any residual product was removed by rinsing with cold water. In the second step, the regenerating shea butter cream was applied to the treated skin, and finally a broad-spectrum sunscreen with SPF 50+ was applied. Participants were instructed to apply the regenerating cream every morning and the glycolic acid-containing fluid every evening as part of their home skincare routine. Those with hyperpigmented lesions were additionally advised to use the depigmenting serum twice daily, applying it only to the discoloured areas. All volunteers were also advised on the importance of consistent photoprotection using sunscreens. The sessions were repeated at 14-day intervals.

### 4.4. Composition of the Tested Formulation

The tested formulation is manufactured according to international patent application WO 2020/260199 A1 [34], which discloses its full qualitative and quantitative composition; the product used in this study corresponds to Example 7 of the patent. Its composition is given in Table 5. The formulation is an aqueous gel with a pH in the range 1.8–2.2 [34], consistent with the approximate pH of 2 stated in Section 4.3. Sepigel™ 305 (Seppic, La Garenne-Colombes, France) is a pre-mixed liquid gelling concentrate; its constituents are listed individually in the manufacturer’s INCI declaration reproduced in Section 4.3. The values in Table 5 represent the nominal manufacturing composition disclosed in the patent; the content of the individual ingredients in the batches used in this study was not independently verified by the investigators. Together with the treatment protocol described in Section 4.3 and the complete INCI listings of the home-care preparations in Supplementary Table S3, this information permits independent replication of the intervention, using either the commercial product or a formulation prepared according to Example 7 of the patent [34].

### 4.5. Photographic Documentation

For each volunteer, facial images were captured using the Fotomedicus version 1 system (Elfo, Łódź, Poland) at three time points: before the first procedure (T0), 2 weeks after the fourth session (T1), and 12 weeks after the fourth session (T2). Clinical photographs were taken in non-polarised light for general documentation, and in cross-polarised light for subsequent image processing and quantitative analysis. The device ensures highly standardised illumination by means of integrated LED light sources located within a fixed photographic dome mounted on a column stand, thereby minimising variability related to ambient or natural light. Cross-polarisation removes specular reflections from the skin surface and sebum film, allowing for more accurate visualisation of pigment alterations within the epidermal and dermal layers. All images were acquired under strictly controlled conditions, including a fixed camera–subject distance, constant camera orientation relative to the face, and identical exposure and white balance settings, which together ensure high reproducibility of measurements. The Fotomedicus platform automatically records metadata (date, time and technical parameters) for every image, facilitating precise longitudinal comparisons and robust quality control of the dataset. The use of a dedicated, calibrated dermatological imaging system rather than consumer-grade cameras markedly reduces colorimetric error (∆E*) and enhances the reliability of objective pigmentation metrics. The photographic data obtained using the Fotomedicus system were subsequently analysed using the grey-level co-occurrence matrix (GLCM) method.

### 4.6. Image Processing and Analysis

In the initial step, colour photographs were captured in the RGB colour model and subsequently separated into their red, green and blue components using ImageJ (version 1.52a). Each original RGB image and its individual channels were then converted to greyscale images in MATLAB (Version 7.11.0.584 (R2010b), 2018). Next, intensity normalisation was performed to extend the dynamic range and enhance image contrast. For the entire dataset of 520 images, this involved identifying the brightest pixel across all images and assigning it an intensity value of 255, and the darkest pixel an intensity value of 0, with all remaining pixels linearly rescaled to grey levels between 0 and 255. Quantitative assessment of pigmented lesions was conducted using GLCM-based algorithms implemented in the same MATLAB environment.

### 4.7. GLCM Analysis

The grey-level co-occurrence matrix quantifies how frequently pixels of a given grey level occur adjacent to pixels of another grey level, and provides second-order texture descriptors whose derivation is described in detail elsewhere [36,37,38]. Hyperpigmented lesions are optically most conspicuous when the contrast between affected and unaffected skin is greatest, so that contrast and homogeneity are the descriptors of interest here. In the present analysis, grey-level relationships were evaluated for horizontally oriented pixel pairs (theta = 0 degrees) separated by one pixel (d = 1); images were analysed at 8-bit depth, giving a co-occurrence matrix of 256 × 256. Contrast, defined in Equation (1), reflects local variation in grey-level intensity; thus, the frequency with which pixels of differing brightness are adjacent; homogeneity, defined in Equation (2), characterises the uniformity of the grey-level distribution within the region of interest.

The GLCM diagram is provided in the Supplementary Material as Figure S2.

(1) $$\sum_{i,j}P_{i,j}{\left(i-j\right)}^{2}$$

where:

i—brightness of the pixel under consideration,
j—brightness of the adjacent pixel.

Regions of interest (ROIs) containing hyperpigmented areas were manually delineated on each patient image and used for subsequent calculations. Homogeneity analysis aimed to characterise the uniformity of grey-level distribution within each ROI, i.e., the degree of evenness in lesion intensity across the entire region. In the adopted model, homogeneity was defined as:

(2) $$\sum_{i,j}\frac{p(i,j)}{1+\left|i-j\right|}$$

where:

i—brightness of the pixel under examination,
j—brightness of the adjacent pixel.

Higher homogeneity values combined with lower contrast values indicate a more uniform skin tone and, consequently, fewer or less pronounced hyperpigmentation changes. Both contrast and homogeneity are expressed in relative (dimensionless) units.

For each image, four ROIs were defined on the face: forehead, left cheek, right cheek, and chin. To evaluate treatment effects within specific facial zones, contrast and homogeneity values were analysed separately for each ROI. To obtain global facial indices, composite measures were calculated using the following weighting scheme:

Face contrast = 0.3 × ROI contrast, forehead + 0.3 × ROI contrast, left cheek + 0.3 × ROI contrast, right cheek + 0.1 × ROI contrast, chin.

Face homogeneity = 0.3 × ROI homogeneity, forehead + 0.3 × ROI homogeneity, left cheek + 0.3 × ROI homogeneity, right cheek + 0.1 × ROI homogeneity, chin.

These composite parameters were then used to describe overall changes in facial pigmentation patterns over time.

### 4.8. Quantitative Assessment of Wrinkle Volume and Skin Relief

Changes in wrinkle volume and skin surface relief were evaluated using the Antera 3D imaging system (Miravex Limited, Dublin, Ireland), which enables the reconstruction of skin micro-relief and the quantitative analysis of topographical parameters. The system was used here to analyse those features of photoaged facial skin that are most clinically relevant—linear and curvilinear depressions corresponding to wrinkles, surface irregularities and micro-relief.

The primary Antera 3D parameter in this study was wrinkle volume, expressed as Volume [mm^3^], calculated along the entire length of each analysed wrinkle segment within the region of interest (ROI). In the context of photoageing, this parameter quantitatively describes the spatial extent of wrinkle depressions, integrating both their depth and width over the full course of the fold. A higher volume value indicates a greater total volume of wrinkle depressions within the analysed region, and thus more pronounced skin relief. A decrease in this parameter following treatment was interpreted as a favourable topographical change, corresponding to a reduction in the overall bulk of the wrinkle depressions and a smoothing of the skin surface.

In addition to wrinkle volume, surface relief indices derived from the three-dimensional reconstruction (roughness-related measures, maximum height differences within the ROI) were analysed as secondary variables describing the heterogeneity of the skin surface micro-relief. From a biological and clinical perspective, higher values of these indices correspond to greater unevenness of photoaged skin, whereas their reduction after treatment indicates smoothing of the micro-relief and a decrease in the prominence of fine lines.

Changes were expressed both as absolute differences and as percentage changes relative to baseline.

### 4.9. Assessment of Skin Viscoelasticity

Skin viscoelasticity was measured with a Cutometer MPA 580 (Courage + Khazaka Electronic GmbH, Cologne, Germany), a suction device that records the vertical displacement of the skin drawn into the probe aperture under a defined negative pressure and derives a set of R parameters from the resulting strain–time curve [39,40]. Measurements were performed with a 2 mm aperture at a negative pressure of 450 mbar, using 3 consecutive cycles of 2 s suction and 2 s relaxation with the probe held perpendicular to the skin and without additional applied pressure. Three ratios were analysed: R2 (Ua/Uf, gross elasticity), R5 (Ur/Ue, net elasticity) and R7 (Ur/Uf, biological elasticity, that is, the proportion of the total deformation recovered elastically) [39,40]. Recordings were obtained from the forehead and from both lateral cheeks at T0, T1 and T2, after at least 15 min of acclimatisation under controlled temperature and relative humidity, and the mean of three consecutive readings per site was used; site values were then averaged to yield a single composite facial value per participant and time point. It should be noted that suction cutometry integrates the mechanical response of the stratum corneum, the viable epidermis and the dermis, and does not identify the tissue compartment or the molecular substrate responsible for an observed change; in particular, epidermal hydration is known to influence the derived parameters [41]. This limitation is addressed in Section 2.2 and Section 3.1.

### 4.10. Assessment of the Degree of Hydration of the Stratum Corneum

Stratum corneum hydration was assessed with a Corneometer CM 825 (Courage + Khazaka Electronic GmbH, Cologne, Germany), which measures the capacitance of the superficial epidermis; capacitance increases with the water content of the stratum corneum, and capacitive measurement is the reference electrical technique recommended by the EEMCO group [42]. Measurements were taken at the same facial sites used for the topographical assessment, at T0, T1 and T2, after at least 15 min of acclimatisation under controlled temperature and relative humidity. The mean of three consecutive readings was recorded for each site and time point.

### 4.11. Assessment of Epidermal Surface Oiliness

Skin surface sebum was quantified with a Sebumeter SM 815 (Courage + Khazaka Electronic GmbH, Cologne, Germany) using the photometric tape method, in which the opacity of a lipid-sensitive tape changes in proportion to the quantity of sebum absorbed and is read by reflectance photometry, in accordance with EEMCO guidance for the in vivo assessment of skin greasiness [43]. The tape was applied perpendicular to the skin for a standardised 30 s at the same facial sites used for the hydration and topographical measurements. Recordings were obtained at T0, T1 and T2 after at least 15 min of acclimatisation under controlled temperature and relative humidity and at least 2 h after the last application of any topical product; the mean of three consecutive measurements was used.

### 4.12. Tolerability Monitoring

The treatment area was inspected by the treating investigator before each of the four applications and at each of the three assessment visits. Participants were asked at every contact whether any cutaneous symptom had occurred since the previous visit, with specific reference to erythema, oedema, desquamation, crusting, pruritus, burning and new pigmentary change. Safety was not a pre-specified endpoint and no structured adverse event form was used; observations were recorded in the participant’s clinical notes.

### 4.13. Subjective Assessment—Pre- and Post-Treatment Questionnaires

In addition to objective instrumental measurements (GLCM analysis, Antera 3D, cutometry, corneometry, sebumetry), a subjective assessment was also carried out using proprietary questionnaires completed by patients before the start of therapy and after completion of a series of treatments with a preparation containing homocysteic acid. Before the first treatment, participants completed an initial questionnaire covering questions regarding their overall satisfaction with the appearance of their face and neck, the presence of aesthetic imperfections (wrinkles, discolouration, loss of firmness, dilated blood vessels, scars, blackheads, etc.) and their location on a schematic drawing of the face. In addition, patients self-assessed the depth of their wrinkles and the severity of skin laxity on a linear Likert scale from 0 to 10, where 0 indicated the absence of wrinkles or very firm skin, respectively, and 10 indicated very deep wrinkles or marked skin laxity.

Upon completion of the treatment series, the patients completed a final questionnaire assessing the subjective change in the appearance of the face and neck on a scale from −5 to +5 (−5 = it is much worse, 0 = I see no significant change, +5 = it is much better). The post-treatment questionnaire also included questions regarding the improvement observed by the patient in specific aesthetic defects (reduction in wrinkles, discolouration, dilated blood vessels, loss of firmness, scars, blackheads), the location of the changes on a schematic diagram of the face, and an assessment of the severity of the changes in terms of wrinkle depth and skin firmness on a scale from −5 to +5 (from marked deterioration to marked improvement). In addition, the overall level of satisfaction with the treatment outcomes and the willingness to continue the treatments were recorded. The post-treatment questionnaire was administered on a single occasion, at the T1 visit two weeks after the fourth session, immediately following the instrumental assessment of that visit. It was not repeated at T2. Self-reported outcomes therefore refer to the two-week time point and are directly time-matched to the instrumental data obtained at that visit. Analysis of the questionnaire results allowed the patients’ subjective assessments to be compared with objective instrumental parameters, thereby enabling a more comprehensive assessment of the clinical efficacy of the chemical skin stimulator in reducing the symptoms of photoageing.

### 4.14. Statistical Analysis

All statistical analyses were performed in Python (version 3.13) using the SciPy library (version 1.17). All tests were two-tailed and the significance threshold was set at α = 0.05.

For each instrumental method, a single composite facial value was derived for every participant at every time point in order to summarise the regional measurements and limit multiplicity. Cutometer viscoelastic ratios (R2, R5, R7) were averaged across the three measured sites. Antera 3D texture indices (texture score, roughness Ra, maximum height), corneometric hydration, and sebumetric sebum output were each averaged across their respective facial sites. Bilateral wrinkle volumes were averaged between sides, yielding four regional outcomes: nasolabial folds, glabellar lines, horizontal forehead lines, and crow’s feet. For the grey level co-occurrence matrix (GLCM) analysis, a weighted whole-face index was computed for contrast and for homogeneity as 0.3 × forehead + 0.3 × left cheek + 0.3 × right cheek + 0.1 × chin, reflecting the relative facial surface area of each region.

The distribution of each variable was examined with the Shapiro–Wilk test. Because several outcomes departed significantly from normality and the sample size was moderate, non-parametric procedures were adopted uniformly across all end points to ensure methodological consistency and robustness. Descriptive data are therefore reported as median and interquartile range (IQR); means ± standard deviations were additionally computed and are given in the text where informative.

The overall effect of time (T0, T1, T2) on each parameter was assessed with the Friedman test, with the effect size expressed as Kendall’s coefficient of concordance (W) and interpreted as small (≈0.10), moderate (≈0.30), or large (≈0.50). When the omnibus test was significant, three pre-specified pairwise contrasts (T0 vs. T1, T0 vs. T2, T1 vs. T2) were evaluated with the Wilcoxon signed-rank test. To control the family-wise error rate, the *p*-values of these three within-parameter comparisons were adjusted with the Holm–Bonferroni procedure. The effect size of each contrast was quantified as the matched-pairs rank-biserial correlation (r), with 0.10, 0.30, and 0.50 denoting small, moderate, and large effects; positive values indicate a higher parameter value at the later time point. Relative changes from the baseline were computed from the medians and expressed as percentages.

The durability of the response was characterised for the surface parameters that peaked at T1 (wrinkle volumes and texture indices). For each participant, the proportion of the peak effect retained at 12 weeks was calculated as (valueT0 − valueT2)/(valueT0 − valueT1) × 100%, restricted to participants who improved at T1. To test the a priori hypothesis that dynamic (mimetic) wrinkles regress more than static wrinkles, the per-participant retention of the static nasolabial folds was compared with the mean retention of the three dynamic regions (glabellar lines, horizontal forehead lines, crow’s feet) using the Wilcoxon signed-rank test. Three additional analyses, performed post hoc in response to the peer review, examined the relationship between the viscoelastic and the hydration response. Per-participant relative changes were computed for each interval and their association was quantified with the Spearman rank correlation coefficient. The influence of age was examined in the same way, by relating age to the baseline value and to the relative change from the baseline of each of the twelve instrumental endpoints, with *p*-values adjusted by the Holm–Bonferroni procedure within each family of twelve; absolute and relative changes were analysed in parallel in order to distinguish a genuine age dependence of the response from a floor effect arising from differing baseline values. The cohort was additionally divided at the median age and the two strata compared with the Mann–Whitney U test. All of these analyses were exploratory, were not pre-specified in the analytical plan and are reported without correction for multiple testing except where stated; the corresponding *p*-values should be interpreted descriptively.

A summary of the statistical procedures applied to each endpoint, with the effect size reported and the method of correction for multiplicity, is provided in Supplementary Table S2.

## 5. Conclusions

A protocol comprising four sessions using a chemical skin biostimulator containing ammonium trichloroacetate, homocysteic acid, phytic acid and citric acid was associated with measurable, statistically significant and largely sustained improvements in instrumentally assessed parameters of mature, photoaged skin across five independent areas of assessment. Skin firmness improved by approximately 24% and was fully maintained, while biological elasticity continued to increase, for three months. This temporal pattern is compatible with, but does not by itself demonstrate, remodelling of the dermal matrix. Wrinkle volume and surface texture improved significantly, peaking after two weeks and then stabilising whilst retaining approximately four-fifths of the peak effect, indicating a lasting structural component superimposed on an early, partially reversible surface effect, which was similar for both static and dynamic wrinkles. Moisture levels in the stratum corneum increased with a delayed but sustained response; sebum secretion gradually decreased; and in participants with hyperpigmentation, skin tone evenness improved and was maintained. The convergence of results from five methodologically distinct measurement techniques, indicating a consistent pattern that can be interpreted in terms of biological mechanisms, is consistent with the concept of a rationally formulated preparation with a multi-directional action, intended to act simultaneously on the skin matrix, the epidermal barrier, oxidative stress and melanogenesis; causal confirmation of this concept will require controlled studies. Within the limitations of an uncontrolled, single-arm study, conducted as part of a comprehensive skincare programme, these results provide encouraging, quantitatively documented observations suggesting that the protocol based on this biostimulator may offer a treatment option with a short recovery time for photoaged skin; they do not by themselves establish the efficacy of the formulation or of any of its components. The pattern of response observed here, namely a durable gain in viscoelasticity, a delayed and sustained increase in hydration, and a surface improvement that peaked early and then partially regressed, is compatible with a formulation acting simultaneously on the dermal matrix and on the epidermal surface. Which component contributes to which effect cannot be determined from this study: the design included no comparison in which any component was omitted or the home-care regimen withheld, although the full quantitative composition of the formulation is known and is reported in Section 3.5 (Table 5). The division of roles proposed in the Discussion is therefore a hypothesis drawn from the published pharmacology of the individual constituents, offered to guide the controlled studies that would be needed to test it. Such studies—incorporating histological analysis and mechanistic endpoints, and ideally comparisons in which individual components are omitted—would determine whether homocysteic acid makes any specific contribution to the observed effects.

The volunteers’ subjective evaluation was strongly and consistently positive: all 25 participants perceived an overall improvement and were satisfied with the treatment, satisfaction with facial and neck appearance rose from 48% to 76%, and significant self-reported improvements were recorded in facial and neck appearance, wrinkle depth and skin firmness (all *p* < 0.001), with high satisfaction ratings for the wrinkle reduction (median 8.2/10) and firming (7.9/10) effects. The concentration of the perceived benefit in the wrinkle and firmness domains, and its regional correspondence with the instrumental measurements, indicates that the subjective experience of the participants is congruent with the objectively documented improvement in skin condition, while the single time point, single-arm design means these self-reported data should be interpreted as corroborative rather than confirmatory. Both sets of data were collected at the two-week assessment; subjective outcomes at twelve weeks were not assessed.

## Figures and Tables

**Table 1:** Baseline characteristics of the cohort before the first application (T0). Values are mean with standard deviation, median with interquartile range and full range. *n* = 25 for all parameters except the GLCM indices, for which *n* = 14.

Parameter	Mean ± SD	Median [IQR]	Range
Age (years)	52.8 ± 10.0	50 [46–62]	39–69
R2, gross elasticity	0.703 ± 0.020	0.709 [0.683–0.718]	0.671–0.733
R5, net elasticity	0.723 ± 0.020	0.729 [0.703–0.738]	0.690–0.753
R7, biological elasticity	0.403 ± 0.020	0.413 [0.382–0.419]	0.375–0.427
Nasolabial fold volume (mm^3^)	10.86 ± 5.78	10.25 [6.76–14.70]	2.84–25.45
Glabellar volume (mm^3^)	6.25 ± 2.78	5.14 [4.04–8.48]	2.27–12.98
Horizontal forehead line volume (mm^3^)	8.26 ± 3.19	8.02 [5.71–9.66]	3.88–16.75
Crow’s feet volume (mm^3^)	5.34 ± 2.34	4.38 [4.03–5.34]	2.34–11.83
Texture score	58.9 ± 10.6	55.0 [52.7–68.7]	44.3–77.7
Roughness Ra (µm)	12.12 ± 2.24	11.57 [10.75–14.06]	8.85–16.19
Maximum height (mm)	0.289 ± 0.071	0.282 [0.256–0.355]	0.141–0.400
Hydration (a.u.)	52.8 ± 8.5	51.9 [49.0–58.7]	35.3–72.7
Sebum (µg/cm^2^)	120.7 ± 14.0	122.6 [114.0–130.2]	87.6–141.8
GLCM contrast	3.18 ± 0.36	3.17 [3.01–3.36]	2.65–3.93
GLCM homogeneity	0.637 ± 0.027	0.638 [0.616–0.658]	0.583–0.673

**Table 2:** Descriptive statistics (median [interquartile range]) and Friedman omnibus test for all instrumental parameters at baseline (T0), 2 weeks after the treatment series (T1), and 12 weeks after the treatment series (T2).

Parameter	*N*	T0	T1	T2	χ^2^(2)	*P*	*W*
Skin firmness—Cutometer							
R2—gross elasticity	25	0.709 [0.683–0.718]	0.879 [0.864–0.895]	0.884 [0.853–0.908]	37.9	<0.001	0.76
R5—net elasticity	25	0.729 [0.703–0.738]	0.903 [0.888–0.922]	0.903 [0.895–0.913]	37.9	<0.001	0.76
R7—biological elasticity	25	0.413 [0.382–0.419]	0.505 [0.491–0.521]	0.516 [0.498–0.540]	49.5	<0.001	0.99
Wrinkle volume—Antera 3D (mm^3^)							
Nasolabial folds (static)	25	10.25 [6.76–14.70]	4.27 [2.63–6.62]	5.26 [3.70–8.08]	48.1	<0.001	0.96
Glabellar lines (dynamic)	25	5.41 [4.04–8.48]	2.43 [1.58–4.60]	3.17 [2.14–5.24]	50.0	<0.001	1.00
Horizontal forehead lines (dynamic)	25	8.02 [5.71–9.66]	3.65 [2.13–4.58]	4.68 [2.90–5.50]	48.1	<0.001	0.96
Crow’s feet (dynamic)	25	4.38 [4.03–5.34]	2.16 [1.94–3.11]	2.63 [2.29–3.35]	50.0	<0.001	1.00
Skin texture—Antera 3D							
Texture score	25	55.0 [52.7–68.7]	22.3 [18.7–25.0]	29.3 [24.0–31.3]	50.0	<0.001	1.00
Roughness Ra	25	11.57 [10.75–14.06]	4.51 [3.83–5.28]	5.99 [5.06–7.13]	50.0	<0.001	1.00
Maximum height	25	0.282 [0.256–0.355]	0.107 [0.090–0.124]	0.141 [0.115–0.161]	50.0	<0.001	1.00
Hydration and sebum							
Hydration	25	51.9 [49.0–58.7]	52.4 [47.1–57.1]	62.6 [60.0–66.6]	37.5	<0.001	0.75
Sebum	25	122.6 [114.0–130.2]	103.4 [99.6–109.8]	93.4 [89.8–97.8]	37.5	<0.001	0.75
Skin tone—GLCM							
Tone contrast (GLCM)	14	3.17 [3.01–3.36]	2.56 [2.37–2.71]	2.55 [2.35–2.70]	21.6	<0.001	0.77
Tone homogeneity (GLCM)	14	0.638 [0.616–0.658]	0.745 [0.730–0.767]	0.750 [0.733–0.768]	24.6	<0.001	0.88

**Table 3:** Relative change from baseline and Holm-adjusted pairwise Wilcoxon signed-rank comparisons for all instrumental parameters.

Parameter	Δ% at T1	Δ% at T2	T0 vs. T1	T0 vs. T2	T1 vs. T2
Skin firmness—Cutometer					
R2—gross elasticity	+23.9	+24.7	<0.001 (+1.00)	<0.001 (+1.00)	0.909 (+0.03)
R5—net elasticity	+23.9	+23.9	<0.001 (+1.00)	<0.001 (+1.00)	0.841 (−0.05)
R7—biological elasticity	+22.3	+24.9	<0.001 (+1.00)	<0.001 (+1.00)	<0.001 (+1.00)
Wrinkle volume—Antera 3D (mm^3^)					
Nasolabial folds (static)	−58.3	−48.7	<0.001 (−1.00)	<0.001 (−1.00)	<0.001 (+0.99)
Glabellar lines (dynamic)	−55.1	−41.4	<0.001 (−1.00)	<0.001 (−1.00)	<0.001 (+1.00)
Horizontal forehead lines (dynamic)	−54.5	−41.7	<0.001 (−1.00)	<0.001 (−1.00)	<0.001 (+0.99)
Crow’s feet (dynamic)	−50.6	−39.8	<0.001 (−1.00)	<0.001 (−1.00)	<0.001 (+1.00)
Skin texture—Antera 3D					
Texture score	−59.4	−46.7	<0.001 (−1.00)	<0.001 (−1.00)	<0.001 (+1.00)
Roughness Ra	−61.0	−48.2	<0.001 (−1.00)	<0.001 (−1.00)	<0.001 (+1.00)
Maximum height	−61.9	−50.1	<0.001 (−1.00)	<0.001 (−1.00)	<0.001 (+1.00)
Hydration and sebum					
Hydration	+0.9	+20.7	0.925 (−0.02)	<0.001 (+1.00)	<0.001 (+1.00)
Sebum	−15.7	−23.8	<0.001 (−0.99)	<0.001 (−0.98)	<0.001 (−0.93)
Skin tone—GLCM					
Tone contrast (GLCM)	−19.2	−19.5	0.003 (−1.00)	0.003 (−1.00)	0.064 (−0.56)
Tone homogeneity (GLCM)	+16.7	+17.6	0.003 (+1.00)	0.003 (+1.00)	0.003 (+0.90)

**Table 4:** Perceived change after the treatment series on −5 to +5 scales, tested against 0 (“no change”) with the one-sample Wilcoxon signed-rank test (*n* = 25).

Domain (+ = Improvement)	*N*	Mean ± SD	Median [IQR]	Positive, %	*p*	*R*
Facial appearance	25	4.27 ± 0.57	4.2 [3.8–4.9]	100	<0.001	+1.00
Neck appearance	25	4.15 ± 0.64	4.1 [3.7–4.7]	100	<0.001	+1.00
Wrinkle depth (+ = shallower)	25	3.67 ± 0.82	3.6 [3.1–4.4]	100	<0.001	+1.00
Skin firmness (+ = firmer)	25	3.49 ± 0.70	3.4 [3.1–3.9]	100	<0.001	+1.00

*R*, matched-pairs rank-biserial correlation. Because all 25 participants reported a change in the same (positive) direction, r reaches its maximum of +1.00 and the *p*-value attains the smallest value achievable for this sample size.

**Table 5:** Quantitative composition of the tested formulation, as disclosed in Example 7 of international patent application WO 2020/260199 A1 [34].

Component	Content (% *w*/*w*)
Ammonium trichloroacetate	33.0
Homocysteic acid	2.0
Citric acid	0.6
Tartaric acid	0.2
Phytic acid	0.2
Glycerol	10.0
Hydrogen peroxide	1.0
Demineralised water	48.0
Sepigel™ 305	5.0
Total	100.0

## Data Availability

The original contributions presented in this study are included in the article/Supplementary Material. Further inquiries can be directed to the corresponding author.

## Supplementary Materials

The following supporting information can be downloaded at: https://www.mdpi.com/article/10.3390/molecules31173038/s1, Figure S1: Perceived change after the treatment series in four self-assessed domains, on a −5 to +5 scale (0 = no change, dashed line; positive = improvement; *n* = 25). Boxes show median and IQR with overlaid individual values. *** *p* < 0.001 versus “no change” (one-sample Wilcoxon signed-rank test); Figure S2: Schematic representation of the GLCM: (a) an example of adjacent pixels in the image, (b) the corresponding pixel configuration in grey-level space, and (c) the counts of pixel pairs for that configuration; Figure S3: Participant flow diagram; Table S2: Summary of statistical procedures applied to each endpoint; Table S3: Complete INCI listings of the home-care preparations; Table S4: Durability of the surface response (former Table 3); File S1: TREND checklist for the reporting of a non-randomised evaluation study; File S2: Questionnaire for a study using PRX plus 1; File S3: Post-treatment questionnaire following treatment with PRX plus 1.
