# Supplementary material for: Instrumental Assessment of a Chemical Skin Biostimulator Combining Ammonium Trichloroacetate and Homocysteic Acid in Photoaged Mature Skin: A Single-Arm Prospective Study
Source: Molecules. 2026 Aug 28;31(17):3038. doi: 10.3390/molecules31173038 (PMC13567254; doi:10.3390/molecules31173038)
Supplement: Supplementary file 1 [file molecules-31-03038-s001.zip › molecules-4456704-supplementary.pdf]

Supplementary materials molecules-4456704

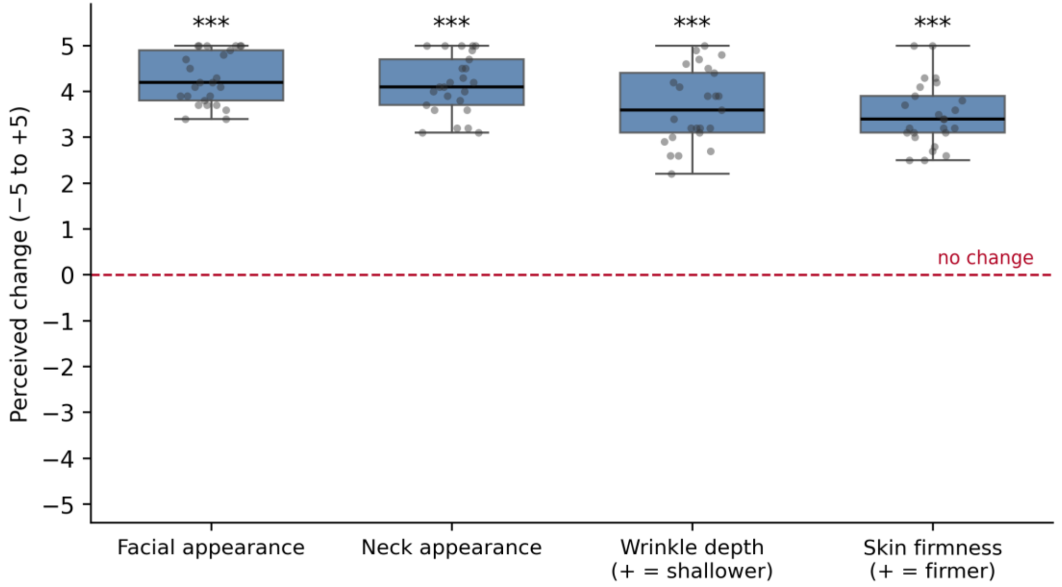

Figure S1. Perceived change after the treatment series in four self-assessed domains, on a −5 to +5 scale (0 = no change, dashed line; positive = improvement; $n = 25$). Boxes show median and IQR with overlaid individual values. ***$p < 0.001$ versus "no change" (one-sample Wilcoxon signed-rank test).

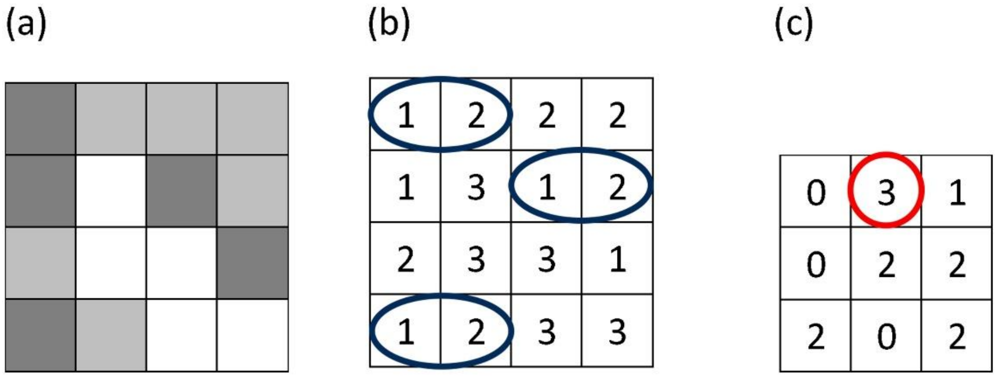

Figure S2. Schematic representation of the GLCM: (a) an example of adjacent pixels in the image, (b) the corresponding pixel configuration in grey-level space, and (c) the counts of pixel pairs for that configuration.

Supplementary Table S2. Summary of statistical procedures applied to each endpoint.

All tests were two-tailed with α = 0.05. The distribution of each variable was assessed using the Shapiro–Wilk test; a uniform non-parametric framework was used because several outcomes departed from normality. Instrumental outcomes are reported as median [IQR].

Endpoint	Analysis set / time points	Primary statistical procedure	Pairwise or additional analysis	Effect size / statistic	Multiplicity adjustment
R2, gross elasticity	n = 25; T0, T1, T2	Friedman test	Wilcoxon signed-rank: T0 vs T1, T0 vs T2, T1 vs T2	Kendall's W; matched-pairs rank-biserial r	Holm–Bonferroni across 3 pairwise contrasts
R5, net elasticity	n = 25; T0, T1, T2	Friedman test	Wilcoxon signed-rank: T0 vs T1, T0 vs T2, T1 vs T2	Kendall's W; matched-pairs rank-biserial r	Holm–Bonferroni across 3 pairwise contrasts
R7, biological elasticity	n = 25; T0, T1, T2	Friedman test	Wilcoxon signed-rank: T0 vs T1, T0 vs T2, T1 vs T2	Kendall's W; matched-pairs rank-biserial r	Holm–Bonferroni across 3 pairwise contrasts
Nasolabial fold volume	n = 25; T0, T1, T2	Friedman test	Wilcoxon signed-rank: T0 vs T1, T0 vs T2, T1 vs T2	Kendall's W; matched-pairs rank-biserial r	Holm–Bonferroni across 3 pairwise contrasts
Glabellar line volume	n = 25; T0, T1, T2	Friedman test	Wilcoxon signed-rank: T0 vs T1, T0 vs T2, T1 vs T2	Kendall's W; matched-pairs rank-biserial r	Holm–Bonferroni across 3 pairwise contrasts
Horizontal forehead line volume	n = 25; T0, T1, T2	Friedman test	Wilcoxon signed-rank: T0 vs T1, T0 vs T2, T1 vs T2	Kendall's W; matched-pairs rank-biserial r	Holm–Bonferroni across 3 pairwise contrasts

Endpoint	Analysis set / time points	Primary statistical procedure	Pairwise or additional analysis	Effect size / statistic	Multiplicity adjustment
Crow's feet volume	n = 25; T0, T1, T2	Friedman test	Wilcoxon signed-rank: T0 vs T1, T0 vs T2, T1 vs T2	Kendall's W; matched-pairs rank-biserial r	Holm–Bonferroni across 3 pairwise contrasts
Texture score	n = 25; T0, T1, T2	Friedman test	Wilcoxon signed-rank: T0 vs T1, T0 vs T2, T1 vs T2	Kendall's W; matched-pairs rank-biserial r	Holm–Bonferroni across 3 pairwise contrasts
Roughness Ra	n = 25; T0, T1, T2	Friedman test	Wilcoxon signed-rank: T0 vs T1, T0 vs T2, T1 vs T2	Kendall's W; matched-pairs rank-biserial r	Holm–Bonferroni across 3 pairwise contrasts
Maximum height	n = 25; T0, T1, T2	Friedman test	Wilcoxon signed-rank: T0 vs T1, T0 vs T2, T1 vs T2	Kendall's W; matched-pairs rank-biserial r	Holm–Bonferroni across 3 pairwise contrasts
Stratum corneum hydration	n = 25; T0, T1, T2	Friedman test	Wilcoxon signed-rank: T0 vs T1, T0 vs T2, T1 vs T2	Kendall's W; matched-pairs rank-biserial r	Holm–Bonferroni across 3 pairwise contrasts
Sebum output	n = 25; T0, T1, T2	Friedman test	Wilcoxon signed-rank: T0 vs T1, T0 vs T2, T1 vs T2	Kendall's W; matched-pairs rank-biserial r	Holm–Bonferroni across 3 pairwise contrasts
GLCM contrast	n = 14; T0, T1, T2	Friedman test	Wilcoxon signed-rank: T0 vs T1, T0 vs T2, T1 vs T2	Kendall's W; matched-pairs rank-biserial r	Holm–Bonferroni across 3 pairwise contrasts
GLCM homogeneity	n = 14; T0, T1, T2	Friedman test	Wilcoxon signed-rank: T0 vs T1, T0 vs T2, T1 vs T2	Kendall's W; matched-pairs rank-biserial r	Holm–Bonferroni across 3 pairwise contrasts
Durability of surface response: wrinkle volume and texture indices	n = 25; T1 peak retained at T2	Per-participant retention: (T0 − T2)/(T0 − T1) × 100; descriptive median [IQR]	Static nasolabial-fold retention vs mean retention of the 3 dynamic regions: Wilcoxon signed-rank test	Matched-pairs rank-biserial r for the retention comparison	None stated for this pre-specified comparison
Self-reported change: facial appearance, neck appearance, wrinkle depth, and skin firmness	n = 25; T1 only	One-sample Wilcoxon signed-rank test against 0 (no change)	Not applicable	Rank-biserial r	None stated
Elasticity–hydration association (post hoc)	n = 25; relative changes over T0–T1, T0–T2, and T1–T2	Spearman rank correlation between relative change in R7 and relative change in hydration	Not applicable	Spearman's ρ	None; exploratory post hoc analysis
Age associations (post hoc)	n = 25; baseline values and change from baseline for 12 instrumental endpoints	Spearman rank correlation of age with baseline value and with absolute and relative change	Age strata (<50 vs ≥50 years): Mann–Whitney U test	Spearman's ρ; no effect size specified for Mann–Whitney U comparisons	Holm–Bonferroni within each family of 12 correlations; stratified comparisons not corrected and descriptive
Safety and tolerability	n = 25; 100 treatment sessions	Descriptive clinical inspection and spontaneous reporting	Not applicable	Not applicable	Not applicable; safety was not a pre-specified endpoint

Abbreviations: GLCM, grey-level co-occurrence matrix; IQR, interquartile range; T0, baseline; T1, 2 weeks after the fourth session; T2, 12 weeks after the fourth session.

Supplementary Table S3. Complete INCI listings of the home-care preparations.

The standardised home-care regimen was used from the first in-office treatment until the 12-week assessment. The lightening serum was supplied only to participants with hyperpigmentation. INCI lists are reproduced in the manufacturer-declared order.

Product	Use in the study	Complete INCI listing
WiQo Nourishing and Moisturizing Cream for Dry Skin	Daytime regenerating cream; all participants (n = 25).	Aqua (Water), Butyrospermum Parkii (Shea) Butter, Propylene Glycol, Paraffinum Liquidum (Mineral Oil), Glyceryl Stearate SE, Hydrogenated Polydecene, Cetyl Alcohol, Bis-PEG-18 Methyl Ether Dimethyl Silane, Simmondsia Chinensis (Jojoba) Seed Oil, Triticum Vulgare (Wheat) Germ Oil, Vitis Vinifera (Grape) Seed Oil, Echinacea

Product	Use in the study	Complete INCI listing
		Angustifolia (Echinacea) Root Extract, Butyl Methoxydibenzoylmethane, Octocrylene, Benzyl Alcohol, Pentaerythrityl Tetra-di-t-butyl Hydroxyhydrocinnamate, Tocopheryl Acetate, Retinyl Palmitate, Helianthus Annuus (Sunflower) Seed Oil, Dehydroacetic Acid, Sodium Gluconate, Parfum (Fragrance), Benzyl Salicylate, Linalool, Limonene, Geraniol, Citral, Citronellol, Hydroxycitronellal, Cinnamyl Alcohol, Hexyl Cinnamal, Amyl Cinnamal, Isoeugenol, Linalyl Acetate, Amyl Salicylate, Citrus Limon Peel Oil, Terpineol.
WiQo Smoothing Face Fluid	Evening smoothing fluid; all participants (n = 25). The formulation contains 8% glycolic acid.	Aqua (Water), Glycolic Acid, Cyclopentasiloxane, Steareth-2, PEG-8 Distearate, Glycerin, Steareth-21, Tocopheryl Acetate, Cetearyl Alcohol, Lecithin, Tocopherol, Ascorbyl Palmitate, Citric Acid, Phytic Acid, Dimethiconol, Sodium Hydroxide, Benzyl Alcohol, Dehydroacetic Acid, Parfum (Fragrance), Linalool, Citronellol, Hydroxyisohexyl 3-Cyclohexene Carboxaldehyde, Hydroxycitronellal, Hexyl Cinnamal.
WiQo Lightening Serum	Applied twice daily exclusively to hyperpigmented lesions; participants with hyperpigmentation (n = 14).	Aqua (Water), Glycerin, Niacinamide, Ethoxydiglycol, Tranexamic Acid, Propanediol, Macadamia Ternifolia Seed Oil, Lactoferrin, Benzyl Alcohol, Ferulic Acid, Sodium Hyaluronate, Thioctic Acid, Sclerotium Gum, Sodium Hydroxide, Aminoethylphosphinic Acid, Dehydroacetic Acid, Tamarindus Indica Seed Polysaccharide, Phytic Acid, Tocopherol, Glycyrrhiza Glabra (Licorice) Root Extract, Hydrogenated Rapeseed Oil, Helianthus Annuus (Sunflower) Seed Oil, Hydrolyzed Algin, Butylene Glycol, Citric Acid.

INCI, International Nomenclature of Cosmetic Ingredients.

Supplementary Table S4. Durability of the surface response (former Table 3).

Proportion of the peak T1 effect retained at 12 weeks (T2) for wrinkle-volume and texture parameters (n = 25).

Parameter	Effect retained at T2, % median [IQR]	Regression %
Nasolabial folds (static)	79 [76–80]	21
Glabellar lines (dynamic)	81 [78–84]	19
Horizontal forehead lines (dynamic)	83 [76–86]	17
Crow’s feet (dynamic)	79 [77–84]	21
Texture score	82 [80–84]	18
Roughness Ra	82 [80–84]	18
Maximum height	82 [79–84]	18

Retention was calculated for each participant who improved at T1 as: ((value at T0 − value at T2) / (value at T0 − value at T1)) × 100. Regression was calculated as 100 − median retention.

Retention of static nasolabial folds did not differ from the mean retention of the three dynamic regions (Wilcoxon signed-rank test: $p = 0.19$; matched-pairs rank-biserial $r = 0.30$). IQR, interquartile range; T0, baseline; T1, 2 weeks after the fourth session; T2, 12 weeks after the fourth session.

Supplementary File S1. TREND checklist for the reporting of a non-randomised evaluation study.

Manuscript: Instrumental assessment of a chemical skin biostimulator combining ammonium trichloroacetate and homocysteic acid in photoaged mature skin: a single-arm prospective study.

Completed according to the Transparent Reporting of Evaluations with Nonrandomized Designs (TREND) Statement. Status: Reported = addressed in the manuscript; Partially reported = addressed incompletely; Not applicable = not relevant to a single-arm uncontrolled design.

Item	Section / topic	TREND descriptor	Status	Location in manuscript / completed checklist response
1	Title and abstract	Information on allocation of units to interventions; structured abstract; target population or study sample.	Reported	Title identifies a single-arm prospective study. Abstract states that 25 women with Fitzpatrick phototype II and clinical signs of facial photoageing received four treatment sessions; no allocation occurred because there was one study arm.
2	Background	Scientific background and rationale; theories used in designing behavioural interventions.	Reported	Introduction (Section 1) provides the biological and clinical rationale for the HoA-containing chemical biostimulator. A formal behavioural theory was not applicable.
3	Participants	Eligibility criteria; recruitment method and setting; settings and locations of data collection.	Reported	Section 4.1 reports inclusion/exclusion criteria, participant characteristics and ethics approval. Sections 4.2 and 4.5–4.11 describe the assessment setting and procedures.
4	Interventions	Content, delivery method, unit and setting of delivery, deliverer, quantity/duration, time span, and adherence activities.	Reported	Sections 4.2–4.4 describe the in-office protocol: three layers per session, four sessions at 14-day intervals, facial areas treated, post-procedure care and home-care regimen. Adherence to home care was verified by structured interview at each visit.
5	Objectives	Specific objectives and hypotheses.	Reported	The final paragraph of Section 1 states the aim: to evaluate non-invasive instrumental changes in photoaged facial skin after the four-session protocol. The single-arm design is described as characterising the treated cohort rather than establishing causality.
6	Outcomes	Clearly defined primary and secondary outcomes; data-collection methods and measurement-quality procedures; validity information where available.	Reported	Sections 4.5–4.13 define the instrumental outcomes (GLCM, Antera 3D, Cutometer, corneometry and sebumetry), questionnaire outcomes and safety observations. Standardised acquisition, acclimatisation, repeated readings, fixed settings and relevant method references are reported.
7	Sample size	How sample size was determined; interim analyses and stopping rules, if applicable.	Reported	Section 4.1 reports that 25 volunteers were enrolled and Sections 2.1 and 4.12 report the analysed sample. No a priori sample-size calculation, interim analysis or stopping rule is reported.
8	Assignment method	Unit and method of assignment to study conditions; measures to minimise bias due to non-randomisation.	Not applicable	The study had one treatment arm only; participants were not allocated to intervention or comparison conditions. This is stated in the title, Abstract, Sections 1 and 3.5–3.6, and the limitations.
9	Blinding (masking)	Whether participants, intervention administrators and outcome assessors were blinded; how blinding was achieved and assessed.	Reported	The study is described as unblinded in the limitations (Section 3.5–3.6). No blinding was implemented in this single-arm prospective study.
10	Unit of analysis	Smallest unit analysed for intervention effects; method if unit of analysis differs from unit of assignment.	Reported	The participant was the unit of analysis. Section 4.12 states that one composite facial value per participant and time point was derived for each instrumental method; no multilevel or cluster adjustment was required.
11	Statistical methods	Methods for primary and additional analyses, missing-data handling, and statistical software.	Reported	Section 4.12 reports Shapiro–Wilk assessment; Friedman tests with Kendall's W; Holm-adjusted Wilcoxon signed-rank contrasts with rank-biserial r; one-sample Wilcoxon tests; Spearman correlations; Mann–Whitney U tests; two-sided $\alpha = 0.05$; Python 3.13 and SciPy 1.17. Supplementary Table S2 maps procedures to endpoints.
12	Participant flow	Numbers through enrolment, assignment, intervention exposure, follow-up and analysis; protocol deviations.	Reported	Section 2.1 states that 25 women completed all three assessments; Section 2.10 reports no withdrawals or discontinuations. Between 29 October 2025 and 1 April 2026, 27 volunteers were assessed for eligibility; two were excluded by the qualifying dermatologist because of health-related contraindications, including skin disease. Twenty-five eligible women were enrolled, received all four treatment sessions, completed T0, T1 and T2, and were included in the main analyses. No participant withdrew, discontinued treatment or was lost to follow-up. The pre-specified GLCM analysis included the 14 participants with baseline hyperpigmentation. A participant-flow diagram is provided as Supplementary Figure S3.
13	Recruitment	Dates defining recruitment and follow-up periods.	Reported	Sections 4.2 and 4.3 define follow-up timing (T0; T1, 2 weeks after the fourth session; T2, 12 weeks after the fourth session) and 14-day treatment intervals. Recruitment, treatment delivery and follow-up took place between 29 October 2025 and 1 April 2026. Four treatment sessions were delivered at 14-day intervals; T1 was performed 2 weeks after the fourth session and T2 12 weeks after the fourth session.
14	Baseline data	Baseline demographic/clinical characteristics; comparison of retained versus lost participants and study sample versus target population.	Reported	Section 2.1 and Table 1 report baseline demographic and instrumental characteristics. No retained-versus-lost comparison was needed because all 25 participants completed follow-up.
15	Baseline equivalence	Baseline equivalence of study groups and methods to control baseline differences.	Not applicable	There was no comparison group in this single-arm study; therefore, between-group baseline equivalence and adjustment for between-group differences were not applicable.

Item	Section / topic	TREND descriptor	Status	Location in manuscript / completed checklist response
16	Numbers analysed	Denominators for each analysis; handling of non-compliers; intention-to-treat status where relevant.	Reported	Tables 1–5 and Supplementary Tables S2 and S4 report n = 25 for most outcomes and n = 14 for GLCM indices. All participants completed the assessments; the study had no allocation or non-complier groups, so intention-to-treat analysis was not applicable.
17	Outcomes and estimation	Results for primary/secondary outcomes, effect estimates and confidence intervals; null/negative findings; causal-pathway tests, if applicable.	Reported	Tables 2–5 and Results Sections 2.2–2.8 provide outcome summaries, p-values and effect sizes (Kendall's W and rank-biserial r), including non-significant results. Confidence intervals are not reported. Mechanistic interpretations are explicitly framed as hypotheses rather than tested causal pathways.
18	Ancillary analyses	Other analyses, including subgroup/restricted analyses; indication of pre-specified versus exploratory analyses.	Reported	Sections 2.2 and 2.9 and Section 4.12 identify the elasticity–hydration and age analyses as exploratory post hoc analyses, and report the GLCM hyperpigmentation subgroup (n = 14).
19	Adverse events	Important adverse events or unintended effects in each study condition.	Reported	Section 2.10 reports transient burning during application and transient erythema resolving within 2–3 h, with no visible peeling, crusting, post-inflammatory hyperpigmentation, serious adverse events, discontinuations or withdrawals. Section 4.12 notes that safety was not pre-specified and no structured adverse-event form was used.
20	Interpretation	Interpretation considering hypotheses, bias, measurement imprecision, limitations, mechanisms/alternatives, implementation/fidelity, and implications.	Reported	Discussion (Section 3) and limitations (Section 3.5–3.6) address the uncontrolled, unrandomised, unblinded design, confounding by home care, limited sample and follow-up, lack of histology and proprietary composition. Alternative explanations and adherence/fidelity are discussed.
21	Generalizability	External validity considering sample, intervention, follow-up, adherence, setting and context.	Reported	Section 3.5–3.6 discusses restricted generalisability: small cohort, women only, Fitzpatrick phototype II, hyperpigmentation subgroup of 14, single-arm design and 12-week follow-up.
22	Overall evidence	Interpretation in the context of current evidence and theory.	Reported	Sections 1 and 3 place the results in the context of published evidence on photoageing, chemical biostimulators, HoA/NMDA signalling and comparator interventions. Conclusions state that the findings are hypothesis-generating and do not establish causality.

Reference: Des Jarlais, D.C.; Lyles, C.; Crepaz, N.; the TREND Group. Improving the Reporting Quality of Nonrandomized Evaluations of Behavioral and Public Health Interventions: The TREND Statement. Am. J. Public Health 2004, 94, 361–366. https://doi.org/10.2105/AJPH.94.3.361.

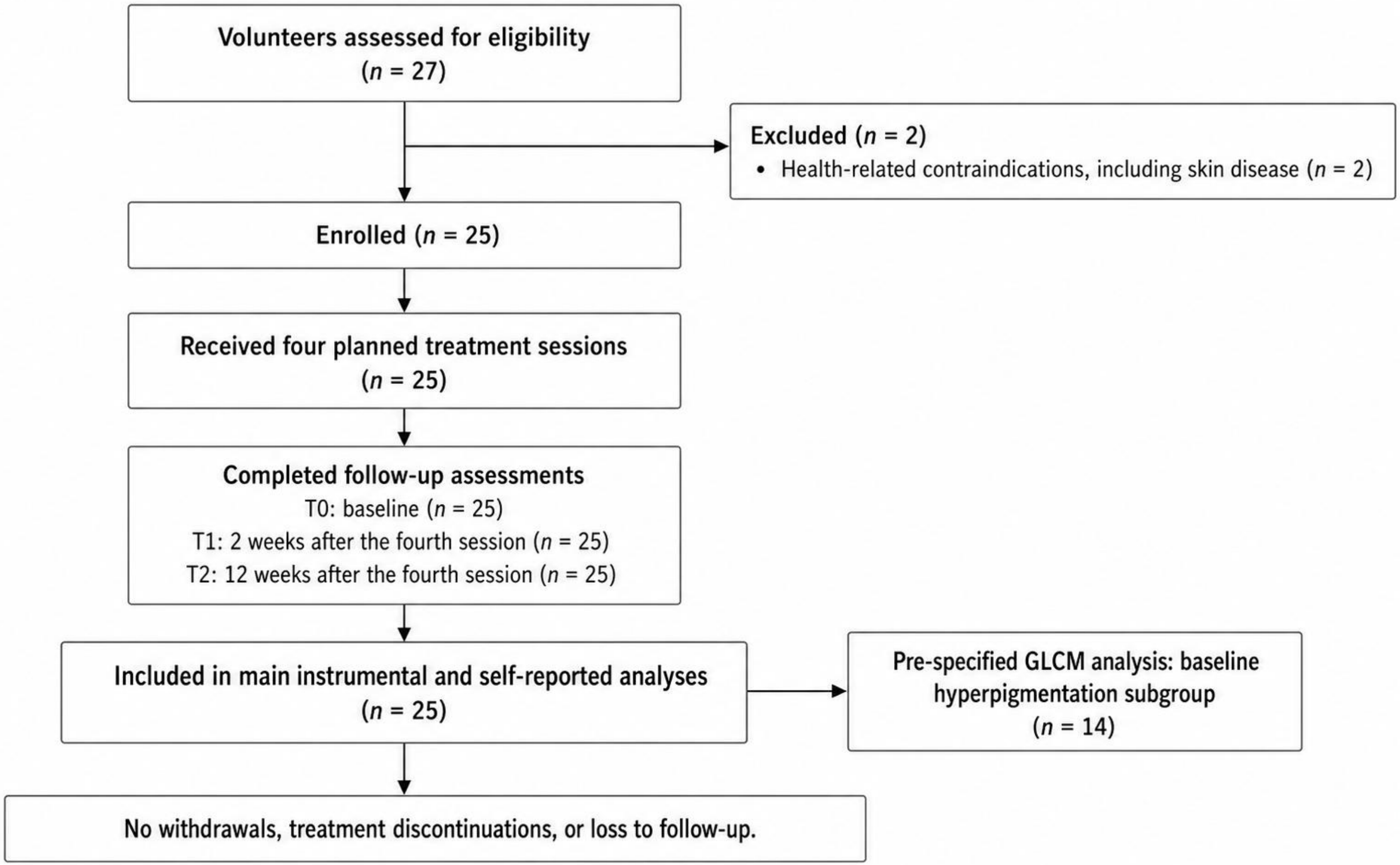

Figure S3. Participant flow diagram.
