# Supplementary material for: Instrumental Assessment of a Chemical Skin Biostimulator Combining Ammonium Trichloroacetate and Homocysteic Acid in Photoaged Mature Skin: A Single-Arm Prospective Study
Source: Molecules. 2026 Aug 28;31(17):3038. doi: 10.3390/molecules31173038 (PMC13567254; doi:10.3390/molecules31173038)
Supplement: Supplementary file 1 [file molecules-31-03038-s001.zip › File S2 - Questionnaire for a study using PRX plus 1.pdf]

File S2 - Questionnaire for a study using PRX plus

First name and surname ………………………………………………………………………………….

Date of birth ……………………….… Date of examination……………………………….

K

1. Are you generally satisfied with the appearance of your face and neck? **YES** **NO**

(PLEASE circle the appropriate option)

Please mark with a line **'I'** on **the top line** to indicate your level of satisfaction with the appearance of your **face,** and **on the bottom line** to indicate your level of satisfaction with the appearance of your **neck** on a scale of

0 = I am very dissatisfied, **10 =** it is perfect

FACE

NECK

0 **10**

2. Do you notice any cosmetic imperfections on your face and/or neck?

YES **NO**

(PLEASE circle the appropriate option)

If so, please circle the aesthetic imperfections you have noticed on the skin of your face and/or neck.

a. Wrinkles
b. Discolouration
c. Discolouration
d. Dilated blood vessels
e. Loss of skin firmness
f. Pigmented lesions (moles)
g. Freckles
h. Scars
i. Blackheads

3. If you notice **any wrinkles**, please mark the areas where they appear on the diagram

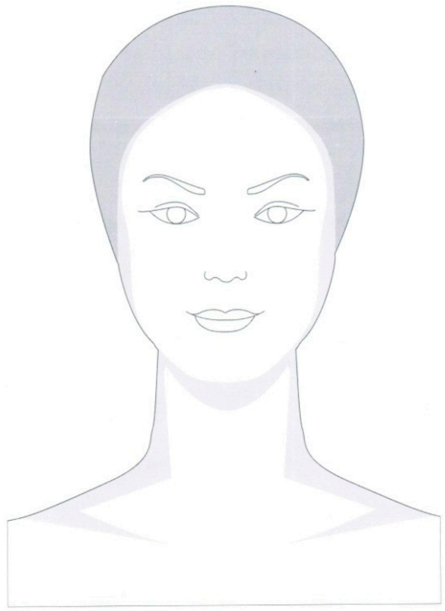

4. If you notice **a loss of skin firmness**, please mark the areas where this occurs on the diagram.

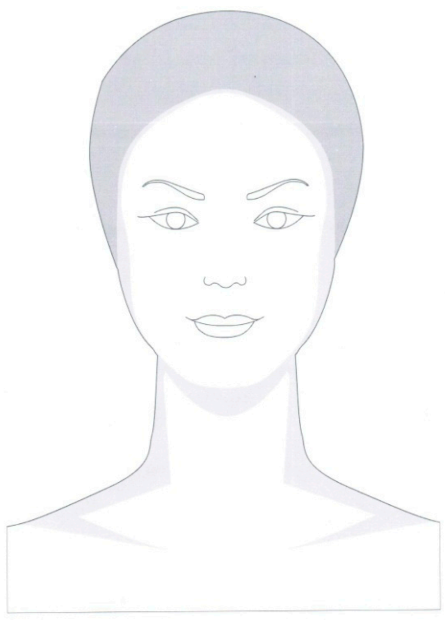

5. If you notice **wrinkles**, please mark the **first** degree of their depth with a line on a scale **of 0 =** I have no wrinkles, **10 =** my wrinkles are very deep.

0 **10**

6. If you notice **a loss of skin firmness,** please mark **with** a line the severity of the changes on a scale **where 0 =** my skin is very firm and **10 =** my skin is very loose

0 **10**

7. Have you ever undergone any cosmetic or aesthetic procedures aimed at reducing wrinkles and/or improve skin firmness?

YES **NO**

(PLEASE circle the appropriate option)

8. If you have undergone anti-wrinkle and/or firming treatments at a beauty or aesthetic clinic, please circle the types of treatments you had.

a. Cosmetic massage
b. Firming face mask
c. Microdermabrasion
d. Cavitation peel/Sonophoresis
e. Radiofrequency treatment
f. Ulthera
g. Cold therapy
h. Chemical peel
i. Needle mesotherapy (hyaluronic acid or vitamin injections)
j. Fillers
k. Botulinum toxin
l. Platelet-rich plasma
m. Laser treatment
n. PRX-T33

9. Are you satisfied with the results achieved following cosmetic/aesthetic treatments?
YES **NO**

(PLEASE circle the appropriate option)

Please mark **with** a line your satisfaction rating with **the wrinkle-reduction** effects achieved through cosmetic/aesthetic treatments on a scale of
0 = I am very dissatisfied, **10 =** the results exceeded my expectations

0 ———————————————— **10**

Please mark **with** a line your level of satisfaction with the **improvement in skin firmness** achieved through cosmetic/aesthetic treatments on a scale of
0 = I am very dissatisfied, **10 =** the results exceeded my expectations

0 ———————————————— **10**
