# Supplementary material for: Instrumental Assessment of a Chemical Skin Biostimulator Combining Ammonium Trichloroacetate and Homocysteic Acid in Photoaged Mature Skin: A Single-Arm Prospective Study
Source: Molecules. 2026 Aug 28;31(17):3038. doi: 10.3390/molecules31173038 (PMC13567254; doi:10.3390/molecules31173038)
Supplement: Supplementary file 1 [file molecules-31-03038-s001.zip › File S3 - Post-treatment questionnaire following treatment with PRX plus 1.pdf]

File S3 - Post-treatment questionnaire following treatment with PRX plus

K

First name and surname ……………………………………………………………………………………….

Date of birth …………………………… Date of treatment……………………………………..

Number of treatments performed……………………………………………………….………

1. Are you generally satisfied with the appearance of your face and neck at present? **YES** **NO**

(PLEASE circle the appropriate option)

Please mark with a line **'I'** on **the top line** to indicate your level of satisfaction with the change in the appearance of your **face**, and **on the bottom line** to indicate your level of satisfaction with the change in the appearance of your **neck** on a scale of

-5 = it is much worse, **0 =** I do not see any significant change, **+5 =** it is much better

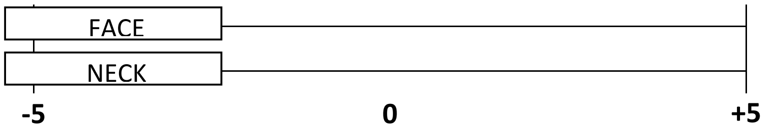

2. After a course of treatments using PRX Plus, have you noticed an overall improvement in your skin's condition? **YES** **NO**

(PLEASE circle the appropriate option)

If so, please circle which aesthetic imperfections on your face and/or neck **have been reduced**.

a. Wrinkles
b. Discolouration
c. Discolouration
d. Dilated blood vessels
e. Loss of skin firmness
f. Pigmented lesions (moles)
g. Freckles
h. Scars
i. Blackheads

3. If you have noticed **a reduction** in your **wrinkles**, please mark the areas on the diagram where the change has occurred.

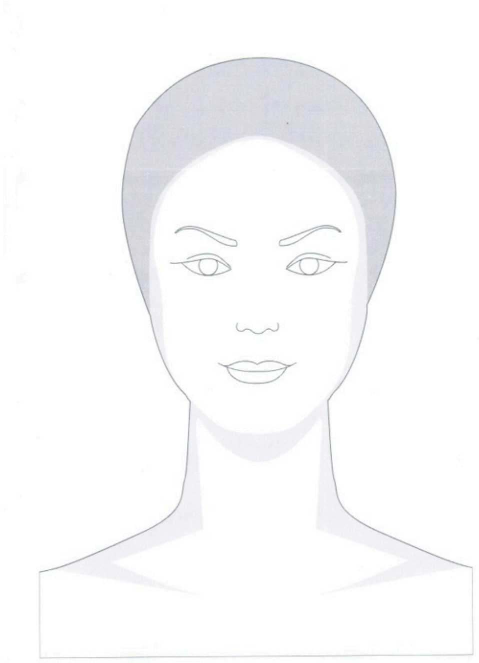

diagram.

4. If you have noticed **an improvement in skin firmness**, please mark the areas where the change has occurred on the

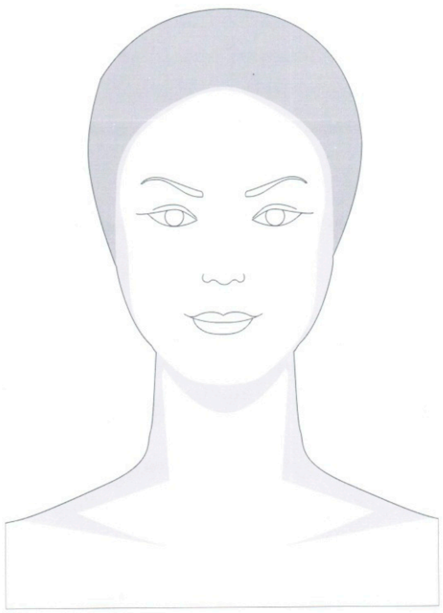

5. If, following a course of PRX plus treatments, you notice **a change in the depth of** your **wrinkles**, please mark **with** a line the extent of the change on the scale: -5 = the wrinkles are much deeper after the treatment, **0 =** I do not see any significant changes, **+5 =** the wrinkles are noticeably shallower.

-5 **0** **+5**

6. If, following a series of PRX plus treatments**,** you notice **a change** in your **skin's firmness,** please mark the degree of change on the scale **with** a line**: -5 =** my skin is definitely more saggy, 0 = my skin's firmness has not changed, **+5 =** my skin is definitely firmer

-5 **0** **+5**

7. Are you satisfied with the results achieved following treatments using the PRX Plus formulation? **YES** **NO**

(PLEASE circle the appropriate answer)

If you are satisfied with the results achieved following treatments using PRX plus:

Please mark **with** a line your satisfaction rating with **the wrinkle-reduction** effects achieved by the treatments on a scale **of 0 =** I am very dissatisfied, **10 =** the results exceeded my expectations

-5 **0** **+5**

Please mark **with** a line your satisfaction rating with the **improvement in skin firmness** achieved through the treatments, on a scale **of 0 =** I am very dissatisfied to **10 =** the results exceeded my expectations

-5 **0** **+5**

8. Would you like to continue treatments using PRX Plus?

YES **NO**

(PLEASE circle the appropriate option)

9. Any other comments regarding the treatment that you would like to make:
